# Human gray matter microstructure mapped using neurite exchange imaging (NEXI) on a clinical scanner

**DOI:** 10.1162/IMAG.a.32

**Published:** 2025-06-12

**Authors:** Quentin Uhl, Tommaso Pavan, Thorsten Feiweier, Gian Franco Piredda, Ileana Jelescu

**Affiliations:** Department of Radiology, Lausanne University Hospital (CHUV) and University of Lausanne, Lausanne, Switzerland; Siemens Healthineers AG, Erlangen, Germany; Advanced Clinical Imaging Technology, Siemens Healthineers International AG, Lausanne, Switzerland; CIBM Center for Biomedical Imaging, Geneva, Switzerland

**Keywords:** diffusion MRI, biophysical models, cortex, human brain, exchange, clinical

## Abstract

Biophysical models of diffusion in gray matter (GM) can provide unique information about microstructure of the human brain, in health and disease. Therefore, their compatibility with clinical settings is key. Neurite Exchange Imaging (NEXI) is a two-compartment model of GM microstructure that accounts for inter-compartment exchange, whose parameter estimation requires multi-shell multi-diffusion time data. In this work, we report the first estimates of NEXI in human cortex obtained on a clinical MRI scanner. To do that, we establish an acquisition protocol and fitting routine compatible with clinical scanners. The model signal equation can be expressed either in the narrow-pulse approximation, NEXI, or accounting for the actual width of the diffusion gradient pulses, SMEX. While NEXI enables a faster analytical fit and is a valid approximation for data acquired on high-performance gradient systems (preclinical and Connectom scanners), on which NEXI was first implemented, SMEX has significant relevance for data acquired on clinical scanners with longer gradient pulses. We establish that, in the context of broad pulses, SMEX estimates were more comparable to previous literature values. Furthermore, we evaluate the repeatability of NEXI estimates in the human cortex on a clinical MRI scanner and show intra-subject variability to be lower than inter-subject variability, which is promising for characterizing healthy and patient cohorts. Finally, we analyze the relationship of NEXI parameters on the cortical surface to the Myelin Water Fraction (MWF), estimated using an established multicomponent T_2_relaxation technique. Indeed, although it is present in small quantities in the cortex, myelin can be expected to decrease permeability. We confirm a strong correlation between the exchange time (*t*_ex_) estimates and the MWF, although the spatial correspondence between the two is brain-region specific and other drivers of*t*_ex_than myelin density are likely at play.

## 1 Introduction

Characterizing the brain’s microstructure has a potentially far-reaching impact on the clinical management of neurodegenerative diseases. Diffusion MRI models offer a unique approach to probing the brain’s tissue architecture, revealing subtle changes that often go undetected with conventional imaging techniques (Jelescu & Fieremans, 2023;Jones, 2010;Pavan et al., 2024). These models can discern alterations in neuronal and glial cells, myelination, and extracellular matrix properties (Alves et al., 2022;Hutchinson et al., 2018;Jelescu, Zurek, et al., 2016;Vestergaard-Poulsen et al., 2007;Wu et al., 2024), which are critical for understanding the progression of diseases such as Alzheimer’s (Dong et al., 2020;Falangola et al., 2013;Gong et al., 2013;Pavan et al., 2023;Tristão Pereira et al., 2021), Parkinson’s (Atkinson-Clement et al., 2017;Sejnoha Minsterova et al., 2020;Zhan et al., 2012), multiple sclerosis (MS) (Liao et al., 2024;Martínez-Heras et al., 2020), first-episode psychosis (Rae et al., 2017), and schizophrenia (Kraguljac et al., 2019;Pavan et al., 2024). The ability of diffusion MRI to reveal and characterize microstructural changes early and accurately could lead to more timely interventions, personalized treatment plans, and better overall patient outcomes.

For white matter, most of the biophysical models of diffusion are variants of a well-accepted general model, the Standard Model of white matter (Novikov et al., 2019).

For gray matter, new models have been recently proposed, complementing the Standard Model either by adding the contribution of cell bodies modeled as spheres (SANDI) (Palombo et al., 2020), of exchange between neurites and extracellular water (NEXI, SMEX) (Jelescu et al., 2022;Olesen et al., 2022), or both (SANDIX) (Olesen et al., 2022). The feasibility of GM models has so far been demonstrated mainly on MRI systems benefiting from high-performance gradients, such as preclinical scanners or human Connectom scanners with maximum gradient amplitudes of ≥300 mT/m (Palombo et al., 2020;Uhl, Pavan, Molendowska, et al., 2024).

Experimental observations of decreasing MR signal with increasing diffusion times in the gray matter suggest exchange as the dominant contributor over restricted diffusion within soma (Jelescu et al., 2022;Olesen et al., 2022). This has implications for the applicability of SANDI, which, by neglecting exchange, is best suited for short diffusion times where the assumption of impermeable compartments remains valid. Moreover, the effects of soma restriction become particularly prominent at high b-values, highlighting the necessity of acquiring high b-value data to effectively probe GM features. Initial attempts to implement SANDI in a clinical setting have been made (Schiavi et al., 2023), demonstrating the growing interest in translating these advanced models to more accessible platforms.

As a parallel attempt to model gray matter, NEXI does not model soma but rather accounts for the inter-compartment exchange, therefore overcoming the need for short diffusion times. However, this comes at the cost of requiring the acquisition of multiple diffusion times. This model characterizes neurites as a collection of randomly oriented sticks, occupying a relative signal fraction*f*. The intra-neurite diffusion is modeled as unidirectional with a diffusivity*D_i_*, while the extra-neurite compartment is modeled as Gaussian isotropic with diffusivity*D_e_*. Water exchange between these two compartments occurs with a characteristic time*t*_ex_.

The NEXI signal equations can be derived under two different assumptions: using the Narrow Pulse Approximation (NEXI) (Jelescu et al., 2022) or accounting for the width of the pulses (introduced as SMEX in (Olesen et al., 2022)). The NEXI and SMEX models, which share identical parameters and describe the same underlying microstructure, diverge only in their handling of the pulse width. NEXI is a valid approximation for data acquired on preclinical scanners (Jelescu et al., 2022;Olesen et al., 2022) and on human Connectom scanners (Lee et al., 2022;Uhl, Pavan, Molendowska, et al., 2024), with gradient pulse duration (δ) values of, e.g., 4 and 9 ms, respectively. However, this assumption is challenged for clinical protocols where δ typically needs to be much longer to compensate for lower gradient amplitude and potentially different stimulation limits.

Here, we propose a clinically feasible NEXI/SMEX protocol and evaluate its performance in characterizing human cortical microstructure non-invasively. First, we evaluate the impact of NEXI versus SMEX on microstructure estimates. Second, similarly to our approach on a Connectom system (Uhl, Pavan, Molendowska, et al., 2024), we evaluate the repeatability of NEXI estimates on a clinical scanner by comparing intra-subject and inter-subject variability using scan-rescan of a cohort of healthy volunteers. Third, we explore for the first time correlations between NEXI-derived cortical microstructure features and complementary techniques such as Myelin Water Imaging. We hypothesized that one of the main drivers of cell membrane permeability (and thus exchange time*t_ex_*) would be myelin content, which is also found in the cortex (Nieuwenhuys, 2013;Palomero-Gallagher & Zilles, 2019) and is expected to reduce permeability. The inter-compartment exchange time could thus serve as an innovative proxy for assessing the density of myelin in gray matter. To robustly quantify myelin using clinical MRI, we used the T_2_-based approach ofPiredda, Hilbert, et al. (2021)to estimate the Myelin Water Fraction (MWF).

## 2 Methods

### 2.1 Theory: NEXI model variants

In both NEXI and SMEX, the signal results from the integration of a kernel around several directions.

$${\bar{S}}_{NEXI}\left(\text{p};q,\;t_{d}\right)=\;\int_{0}^{1}K\left(q,\;\text{g},\;t_{d};\text{p},\text{n}\right)d{\left(\text{g}.\text{n}\right)}^{2}$$ (1)

where$\text{p}=\left[t_{ex},\;D_{i},\;D_{e},\;f\right]$are the microstructure parameters to fit,**n**are the neurite orientations,*q*is the wave vector along direction**g**(Jelescu et al., 2022;Olesen et al., 2022).

Each method has its own way of deriving the kernelK. NEXI, employing the Kärger model approximation (Kärger, 1985), simplifies the complex exchange dynamics by treating diffusion encoding pulses in the Pulsed Gradient Spin Echo (PGSE) design as instantaneous Dirac pulses, separated by a duration Δ. This approximation facilitates a more straightforward analytical solution but at the cost of accuracy. A correction term is introduced, however, by considering the diffusion time to be t_d_= Δ-δ/3, instead of Δ (Moutal et al., 2018).

On the other hand, SMEX adopts a more theoretically comprehensive stance by numerically integrating the system over the duration of the gradient pulses, acknowledging the real pulse width. Its signal was calculated using the Initial Value Problem (IVP) solver (*solve_ivp*from scipy.integrate (Virtanen et al., 2020)). While this approach enhances model fidelity to actual experimental conditions, it demands higher computational resources and faces challenges in numerical stability and precision. An efficient way to speed up the fit is to solve the equation on the segment [δ, Δ] whose solution is:

$$S\left(t=\Delta \right)=exp\left(\left(\Delta -\;\delta \right).\left(R-q^{2}D\left(\text{g}.\text{n}\right)\right)\right).S\left(t=\delta \right)$$ (2)

where*R*and*D*are respectively the rate and diffusivity matrices of the generalized rate equation (Ning et al., 2018), as well as to jointly calculate the signal and its analytical Jacobian and to use the tensor formalism. These formulae are provided in our code repository (https://github.com/Mic-map/graymatter_swissknife).

### 2.2 Experimental

#### 2.2.1 Participants

The study was approved by the ethics committee of the canton of Vaud, Switzerland (CER-VD). Written informed consent was obtained from all participants. Data were acquired in 11 healthy adults (6 M, 26.9 ± 1.3 years old). Each participant was scanned twice (delay between scans: 65 ± 29 days).

#### 2.2.2 Data acquisition

All data were acquired on a 3T MRI system with 80 mT/m gradients (MAGNETOM Prisma, Siemens Healthineers AG, Forchheim, Germany). An anatomical reference was acquired using an MP-RAGE sequence (1-mm isotropic resolution, FOV = 224 x 240 mm^2^, 256 slices, TI/TR = 900/1760 ms). Diffusion-weighted images were acquired using a PGSE Echo-Planar Imaging (PGSE EPI) research sequence with b-values = 1.00 and 2.00 ms/µm² at diffusion times Δ = 28.3 and 36.0 ms, b-values = 1.00, 2.00, 3.20, 4.44 ms/µm² at Δ = 45.0 ms, and b-values = 1.00, 2.00, 3.20, and 5.00 ms/µm² at Δ = 55.0 and 65.0 ms, 20 directions per shell, in addition to one b = 0 ms/µm² images per Δ and one b = 0 ms/µm² with reversed EPI phase encode direction for susceptibility distortion correction. Other parameters were fixed: δ = 16.5 ms, TE/TR = 100 ms/5 s, FOV = 256 x 256 mm^2^, matrix: 128 x 128, 64 slices, 2-mm isotropic resolution, partial Fourier = 6/8, GRAPPA = 2, SMS = 2. The total scan time for dMRI was 27 min. The various diffusion times were acquired in scrambled order to avoid any confounds from scanner drift: Δ = 65, 28.3, 45, 36 and 55 ms. Multi-echo T_2_data were collected using a 3D multi-echo accelerated gradient and spin echo (GRASE) research sequence (Piredda, Hilbert, et al., 2021) with voxel-size = 1.8 x 1.8 x 1.8 mm^3^; ΔTE/N-echoes = 10.94ms/32; TR = 5 s, matrix-size = 112 x 128 x 76.

#### 2.2.3 Diffusion data preprocessing

While each diffusion time was acquired in a separate acquisition run, all multi-shell multi-diffusion time data (N = 325 volumes) were pooled together for pre-processing. Pre-processing included Marchenko-Pastur principal component analysis (MP-PCA) magnitude denoising (Veraart et al., 2016), Gibbs ringing correction (Kellner et al., 2016), distortion and eddy current correction (Andersson & Sotiropoulos, 2016). A separate MP-PCA denoising of b = 0 and b = 1 ms/µm² images (N = 112 volumes) was used to extract an unbiased noisemap, σ, from high SNR data, to be used in the Rician mean correction. For NEXI/SMEX estimation, data were averaged over directions (powder-average, using the arithmetic mean) and normalized by the mean value of the b = 0 volumes of each respective diffusion time sequence.

#### 2.2.4 Cortical thickness and ROI parcellation

Cortical thickness was extracted from anatomical MPRAGE images using FastSurfer (Henschel et al., 2020). Grey matter region of interests (ROIs) from the Desikan-Killiany-Tourville (DKT) atlas (Klein & Tourville, 2012) were segmented on the anatomical MPRAGE image using FastSurfer and transformed into diffusion native space using linear registration of distortion-corrected b = 0 images to MPRAGE images. The cortical ribbon was segmented by merging the gray matter ROIs obtained with the DKT atlas. Mean values of NEXI parameters, MWF, and cortical thickness were calculated for each ROI. The spatial distribution of these measures averaged across subjects was examined using inflated brain surfaces obtained from Connectome Workbench (Marcus et al., 2011).

#### 2.2.5 NEXI and SMEX parameter estimation and comparison

In this work, the expectation value of the Rician noise floor is consistently incorporated directly into the model fit, as described in (Uhl, Pavan, Molendowska, et al., 2024). The Rician floor markedly affects low-SNR images, i.e. high b-value images in our case. The Rician scale σ (which determines the Rician floor as$\sqrt{\frac{\pi }{2}}\sigma$) was fixed to the noise standard deviation estimated using MP-PCA during preprocessing.

The NEXI/SMEX model variants were fit to the experimental data by Nonlinear Least Squares (NLS) using the L-BFGS-B algorithm and*minimize*function (from*scipy.optimize*(Virtanen et al., 2020)) with a tolerance of 1e-14. The bounds specified for the optimization were [1 - 150] ms for*t*_ex_, [0.1 - 3.5] µm²/ms for the two diffusivities and [0.1 - 0.9] the fraction*f*. The initialization had the constraint that*D*_i_>*D*_e_(Dhital et al., 2019;Howard et al., 2022;Kunz et al., 2018).

An initial grid search was applied before the NLS to find an optimal starting point. The parametric maps of NEXI and SMEX are compared on the inflated brain surface, as well as the averages of the parameter values in each ROI.

For 200,000 voxels, approximately the size of a full-brain mask, the computation time using 48 CPUs in parallel on a high-performance cluster is 5 h and 25 min for SMEX vs. 45 min for NEXI.

To compare the models’ goodness-of-fit to our experimental data, we used the corrected Akaike Information Criterion (AICc) (Akaike, 1973). A lower AICc suggests a better fit.

While our implementation relies on solving the SMEX model using direct numerical integration or analytical shortcuts for specific time intervals, recent work by (Olesen et al., 2022) has demonstrated an efficient alternative: replacing the forward model with a neural network trained on ODE-based signal solutions. This surrogate is used only for signal prediction and still relies on nonlinear least squares for parameter estimation. By doing so, the approach preserves interpretability and avoids the risks associated with full machine-learning-based inversion. Although not used in this study, such acceleration strategies appear promising and warrant further validation under realistic experimental and noise conditions.

### 2.3 Performance evaluation

#### 2.3.1 Suitability of the NEXI/SMEX models vs. SANDI

The key signature of exchange is decreasing signal with increasing diffusion time, at a fixed b-value. On the contrary, restriction (e.g. in soma) translates into a higher signal with increasing diffusion time (Jelescu et al., 2022;Olesen et al., 2022). To verify the sensitivity of clinical-grade data to exchange, we tested the downward slope of signal versus diffusion time at various b-values (primarily b = 2 ms/µm^2^, due to the availability of five diffusion times) and various ROIs.

We also compared the goodness of fit and plausibility of parameter estimates between NEXI, SMEX and SANDI. For SANDI, we fit the model on two data subsets: either the entire available data (multi-shell, multi-t) or on a single diffusion time (as implemented inPalombo et al. (2020))chosen to be Δ = 45 ms, as the shortest possible to limit exchange effects, yet with b-values high enough to probe restriction. SANDI was estimated using both our NLS implementation (https://github.com/Mic-map/graymatter_swissknife) or the online estimator (https://github.com/palombom/SANDI-Matlab-Toolbox-Latest-Release).

#### 2.3.2 Noise propagation using synthetic signals generated from experimental distributions

The effect of pulse width on Kärger model estimates, assuming isotropic exchanging compartments, has been previously explored using Monte Carlo simulations (Nilsson et al., 2010). Here, to assess the propagation of noise in our estimates and how the NEXI model handled signals generated using wide gradient pulses, we used synthetic ground truth signals drawn from the parameter distributions obtained throughout the cortex with SMEX. We selected 10,000 voxels whose SMEX estimates converged without hitting any bound, and whose diffusivities respected*D_i_*>*D_e_*. We then added Rician noise, with 20 repetitions to mimic the number of directions in the acquisition protocol, averaged them as done for the powder-average and estimated the NEXI and SMEX parameter values using the same fitting procedure as for experimental data. To verify that the model performance was not biased by the choice of ground truth distributions, we additionally simulated synthetic signals from flat distributions spanning plausible parameter ranges (*t_ex_*∈ [1, 40] ms,*D_i_*∈ [2.5, 3.5] µm²/ms,*D_e_*∈ [0.5, 1.5] µm²/ms,*f*∈ [0.2, 0.7]), inferred from the full voxelwise estimates.

#### 2.3.3 Repeatability and brain region-specific patterns

Intra-subject versus inter-subject variability was assessed on average GM mean ROI estimates obtained by the NEXI variants. The different correlation coefficients between estimates from subjects and between sessions of the same subjects are also examined.

### 2.4 Myelin comparison

#### 2.4.1 Myelin water fraction estimation

MWF was estimated using the multicomponent T_2_toolbox (https://github.com/ejcanalesr/multicomponent-T2-toolbox). Recent benchmark of non-parametric T_2_relaxometry methods (Canales-Rodríguez et al., 2021) recommended the use of either χ²-I or L-curve-I to fit the T_2_distribution, depending on the noise level. A comparison of the sharpness of the cut-off between the T_2_distribution lobes with each technique led us to choose χ²-I (seeSupplementary Fig. S1for an example). MWF was therefore estimated using the χ²-I non-parametric T_2_relaxometry method for myelin water quantification (Canales-Rodríguez et al., 2021).

#### 2.4.2 Relationships between MWF and NEXI/SMEX parameters

We calculated the correlation between NEXI or SMEX parameter estimates and MWF across ROIs, averaged over subjects. The cortical surface maps of*t_ex_*and MWF are visually compared.

#### 2.4.3 Relationships between MWF and DKI parameters

DTI/DKI scalar parameters (mean, axial, radial diffusivity and kurtosis) were estimated using the DKI weighted linear least squares estimation (Veraart et al., 2013) in DESIGNER (Ades-Aron et al., 2018). We calculated the correlation between these estimates and MWF across ROIs, averaged over subjects, and compared the correlation coefficients obtained with those of NEXI/SMEX.

#### 2.4.4 Relationships between cortical thickness and NEXI/SMEX parameters

NEXI/SMEX parameters estimates measured in a GM voxel might be influenced by Partial Volume Effects (PVE) with neighboring WM or CSF. As areas of thinner cortical thickness may be more affected by PVE due to the relatively large DWI voxel size (2 mm), we therefore also evaluated potential correlations between the cortical thickness and the model parameter estimates across ROIs.

## 3 Results

### 3.1 Suitability of NEXI/SMEX to fit clinical-grade data of the human cortical ribbon

Experimental decay curves averaged over the entire cortical ribbon and all subjects show the expected decrease in signal with longer diffusion times, at each b-value (Supplementary Fig. S2). Both NEXI and SMEX fit the data well, across the entire range of b-values.

When comparing signal decay with longer diffusion times between gray matter and white matter, we found a 3.4 % decrease between the shortest and longest diffusion times at b = 2 ms/µm² in gray matter, compared to 1.1 % in white matter.

To demonstrate sensitivity to exchange at the individual and local level, we conducted a focused analysis on a single example subject/scan from our dataset. We analyzed the signal time-dependence at b = 2 ms/µm², as this b-value provided the largest number of distinct diffusion times (n = 5). We analyzed 62 cortical ROIs within this subject.

**Fig. 1.**
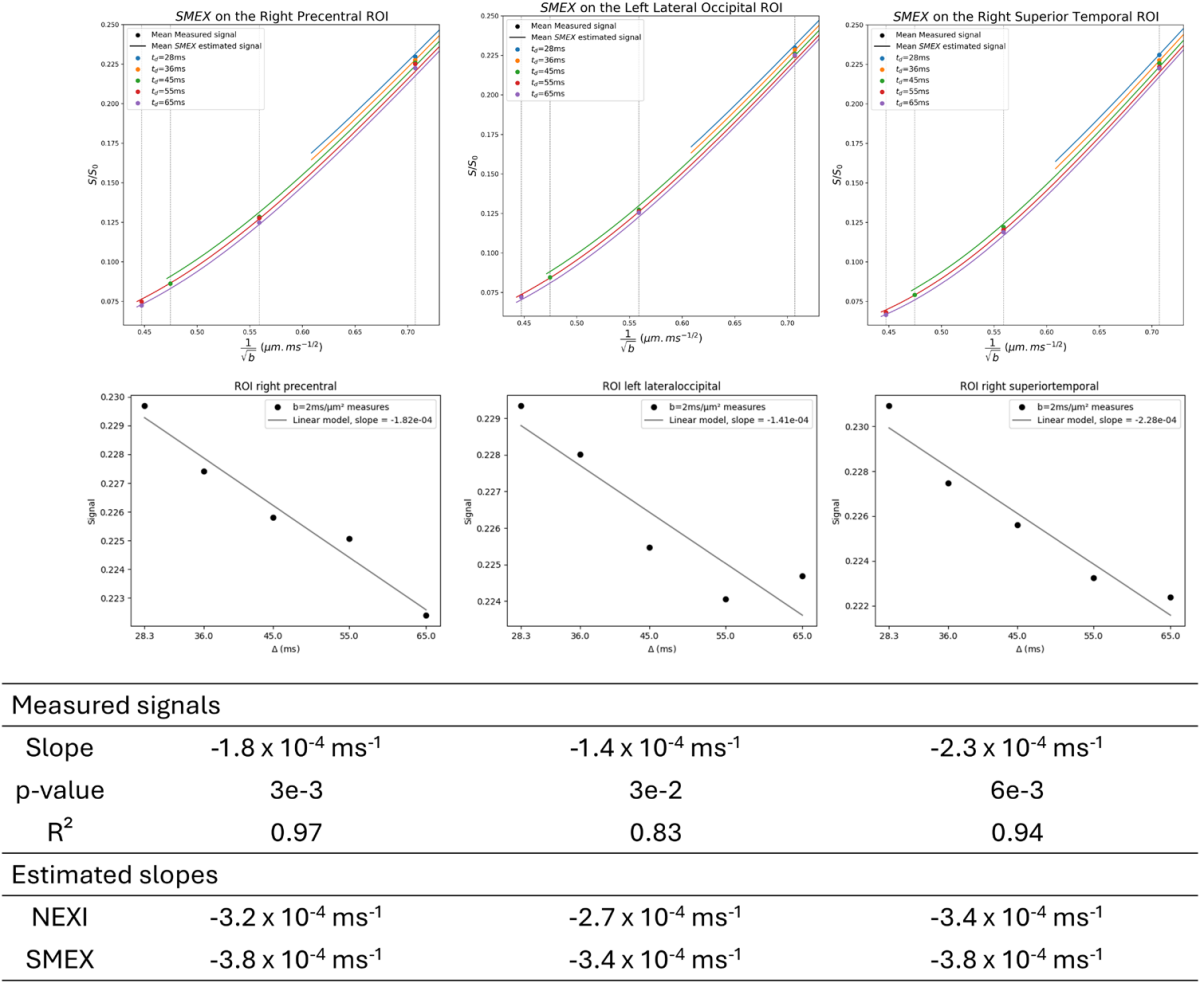
Single-subject ROI analysis in the right precentral, left lateral occipital, and right superior temporal ROI. Mean estimated SMEX signal curves vs. mean measured signal in each ROI are shown as well as downward trend of the signal at b = 2 ms/µm² depending on the diffusion time. The signal is significantly decreasing in most of the ROIs like the three that are shown at b = 2 ms/µm². Both NEXI and SMEX fits predict a faster signal decay at this specific b-value, as the fits are performed over the entire b–t_d_range, making them sensitive to the other points at different b-values.

In 51 out of 62 ROIs, we measured a negative slope for signal as a function of diffusion time, with a p-value <0.05, indicating a statistically significant downward trend. An additional 10 ROIs also showed a negative slope, although these did not reach statistical significance (p > 0.05). These ROIs were predominantly located in deeper brain regions characterized by higher partial volume effects, such as the fusiform, pericalcarine, and parahippocampal gyri.Figure 1shows representative examples of three ROIs and their fits.

Furthermore, we examined the signal slopes at b = 3.2 ms/µm² and b = 1 ms/µm². At b = 3.2 ms/µm², all ROIs except one exhibited a negative slope. However, due to the limited number of Δ values (n = 3), only 13 of these ROIs reached statistical significance (p < 0.05). At b = 1 ms/µm², 57 out of 62 ROIs showed a negative slope, but only 16 achieved statistical significance (p < 0.05), despite having 5 distinct Δ values. This is consistent with the Gaussian phase approximation at low b-values, where the model assumes, in principle, no diffusion time-dependence (i.e., the initial slopes should be similar for all diffusion times).

Finally, when fit to a single diffusion time, SANDI predicted the experimental signal well for that diffusion time but not for the others (and was worse for the machine learning implementation than for the NLS implementation), while a SANDI fit of all b-values and diffusion times jointly yielded modest fit quality to the data, and visibly poorer than NEXI/SMEX, with AICc values to be compared to NEXI/SMEX ones fromTable 1here below (Supplementary Fig. S3). Notably, neurite fractions were unrealistically low and soma radii too large.

**Table 1.** Modes and confidence intervals of NEXI estimates across DKT ROIs.

	* t _ex_* (ms)	*f*	* D _i_* (µm²/ms)	* D _e_* (µm²/ms)	AICc
NEXI	41.1 [15.5 - 66.7]	0.51 [0.41 - 0.61]	2.61 [2.03 - 3.20]	1.59 [1.18 - 2.00]	-91.5 [-98.5, -84.4]
SMEX	36.8 [17.6 - 58.0]	0.42 [0.34 - 0.50]	2.45 [1.99 - 2.90]	1.23 [0.88 - 1.61]	-87.7 [-93.4, -80.0]

The mode and confidence intervals of the corrected Akaike Information Criterion (AICc) are also given.

### 3.2 NEXI/SMEX parameters in the human cortical ribbon

**Fig. 2.**
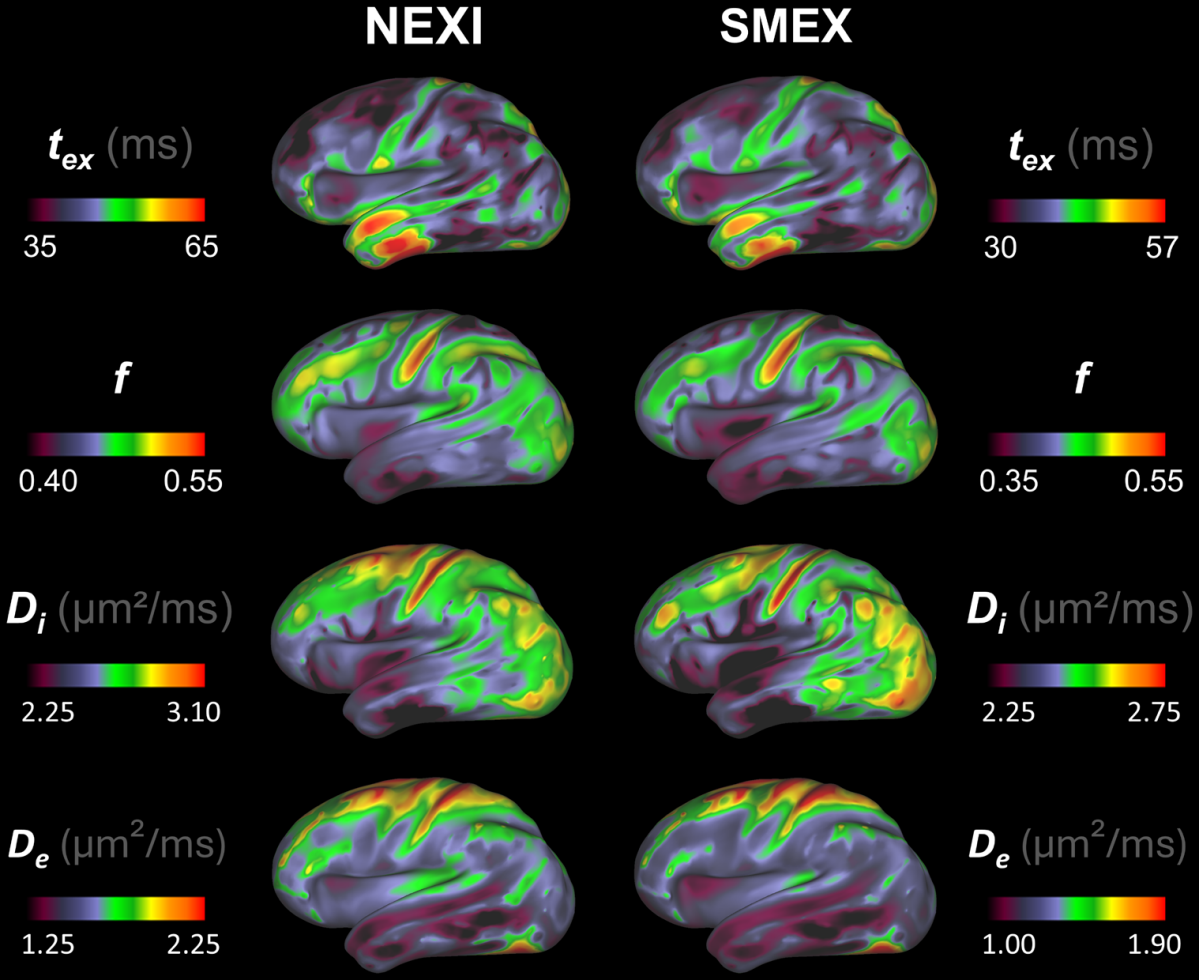
Projection onto cortical surface of NEXI and SMEX maps averaged across subjects and sessions. While the overall patterns remained consistent between models, there is a noticeable shift in scale from one model to the other. As compared to other brain areas, higher values of*f*,*D_i_*, and*D_e_*are recorded around the central sulcus, contrasting with lower values in the anterior temporal lobe.

We report the first estimates of gray matter microstructure in the human brain obtained using NEXI and SMEX on a clinical scanner, illustrated as inflated brain surfaces (Fig. 2). Example axial slices are shown inSupplementary Figure S4. As anticipated, the observable patterns across brain regions are identical between the two implementations, with the only significant difference being a slight shift in the scales – i.e. somewhat lower estimates of*t*_ex_,*f*,*D*_i_and*D*_e_for SMEX vs. NEXI. Both implementations however yield a*t_ex_*between 30 and 60 ms in the cortex, which is on the order of the previous estimate of 42 ms on the Connectom scanner (Uhl, Pavan, Molendowska, et al., 2024).

The brain surface maps consolidate some patterns described in (Uhl, Pavan, Molendowska, et al., 2024), notably along the central sulcus and the anterior temporal lobe. We further observe a pattern of longer*t_ex_*in the temporal lobe, reaching values exceeding 50 ms, paired with a lower*D_i_*estimate.

There are some notable differences with initial estimates on the Connectom scanner (Uhl, Pavan, Molendowska, et al., 2024). For example, higher*D_e_*in the insula has disappeared.

The distribution of each parameter across the cortical ribbon voxels is plotted inFigure 3A. We note a bimodality of NEXI for the estimation of fraction*f*, but also more estimates close to the upper bounds. Thus, the main modes of*t_ex_*,*f*and*D_e_*are located at lower values than the range displayed for the brain surfaces (Fig. 2) for NEXI, due to smoothing across this bi-modal distribution. Notably, the*t_ex_*mode is located around 2 ms for NEXI and 11 ms for SMEX, the latter being closer to the values reported in human cortex to date using Connectom scanners and shorter gradient pulses (Chan et al., 2024;Uhl, Pavan, Molendowska, et al., 2024).

**Fig. 3.**
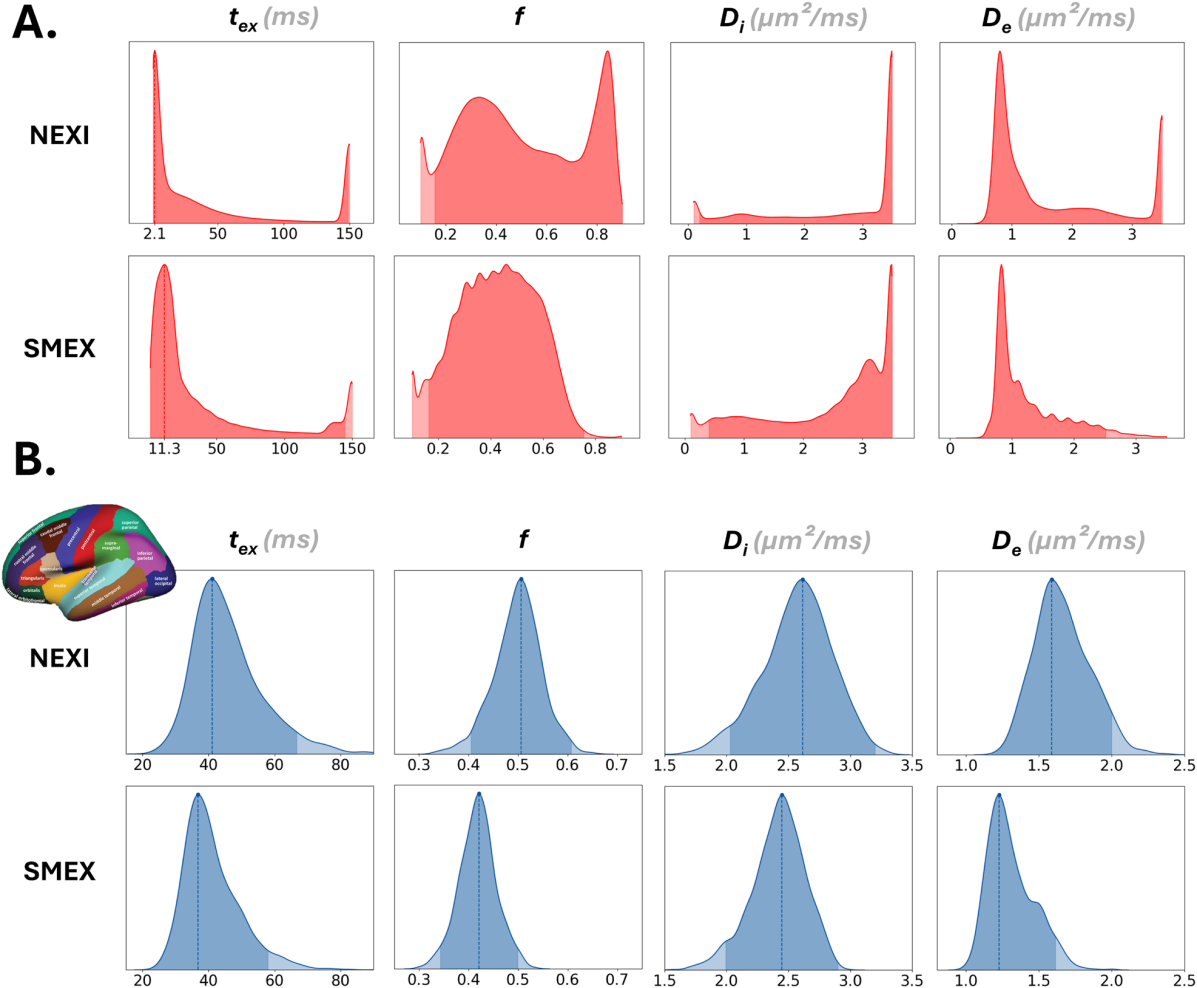
(A) Parametric distributions across voxels in the cortical ribbons from NEXI and SMEX estimations. The NEXI model most often gives values at the boundaries and produces a bimodality in*f*, which completely disappears with the other NEXI variant. The SMEX fit shows comb-like peaks that result from the combination of the ODE solver’s imprecision with the initial grid-search. (B) Distributions of ROI means from the DKT atlas (top-left illustration), from NEXI (top row) and SMEX (bottom row) estimations. The values obtained with SMEX are lower than those with NEXI.

To gain insight at the ROI-level, we also examine the NEXI parameter distributions across the 68 ROIs of the DKT atlas averaged within each ROI and over all subjects (Fig. 3B). These distributions yield well-defined modes, which we report inTable 1, with the 95% confidence intervals obtained by integrating around the mode. The distribution of ROI means (Fig. 3B) looks very different from the distribution of pooled voxel values (Fig. 3A) likely because all the cortical ROIs are similarly affected by outlier voxels vs. voxels with more sensible estimates. Outlier voxel values are possibly due to partial volume effects with CSF or sub-cortical WM. The ROI-averaged values are in better agreement with the smoothed brain surface values inFigure 2.

Finally,Table 1also reports the mode and confidence intervals of the AICc of the two models. The full distribution of AICc values across the DKT ROIs is shown inSupplementary Figure S5. A Wilcoxon signed-rank test was performed to compare the distributions of scores, which showed that NEXI AICc is significantly lower than SMEX AICc, p < 0.0001. These results suggest that the signals derived from NEXI estimates are surprisingly closer to the experimental signals.

### 3.3 Fitting performance evaluation

#### 3.3.1 NEXI noise propagation: fitting synthetic ground truth signals

Estimation performance on the synthetic dataset is shown inFigure 4. As the underlying ground truth is generated considering the actual diffusion gradient pulse width, the accuracy of SMEX estimates is good, while NEXI estimates are naturally more biased. For both variants, the accuracy on*t_ex_*deteriorates with longer times, which is due to the limited diffusion time range sampled, and the precision is poorest on*t_ex_*and*D_i_*, as previously reported (Jelescu et al., 2022). NEXI biases reflect differences in the distributions obtained on the experimental data. For example,*t_ex_*values are underestimated while*D_i_*is overestimated.*D_e_*estimates are relatively unbiased in the vicinity of 1 µm²/ms, echoing the almost identical estimation modes of these two models on our experimental data. However, for*f*, NEXI outputs are understimated, while this model variant produced a higher mode for*f*than SMEX in the experimental data. These findings were further supported using synthetic data generated from uniform parameter distributions within plausible ranges, rather than distributions derived from SMEX estimates, to minimize potential circularity. While estimation errors were generally reduced for NEXI under this configuration, particularly for*f*and*D_e_*, the overall trends remained consistent, with SMEX providing more accurate estimates across most parameters (Supplementary Fig. S6). Under equivalent data and fit, but in a narrow pulse configuration with δ reduced to 4 ms, the performance of the two models is more similar. SMEX shows more bias than NEXI in the estimation of*f*, somewhat poorer precision in the estimation of*t*_ex_, but retains better performance in estimating*D*_i_. (Supplementary Fig. S7).

**Fig. 4.**
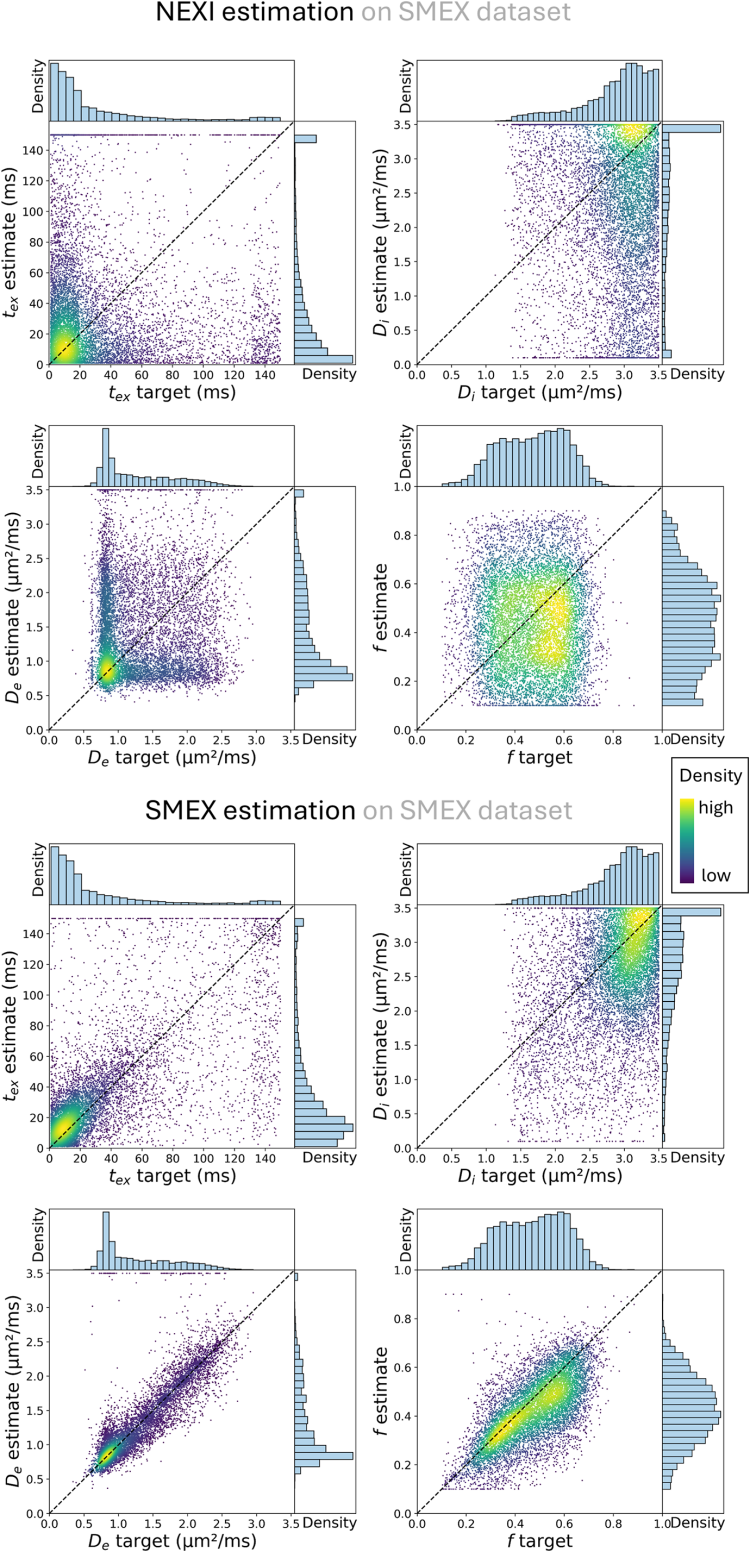
Scatter plot of NEXI and SMEX parameter estimates from synthetic SMEX signals generated using the experimental estimates from SMEX as ground truth, and experimental Rician noise levels.*t_ex_*and*D_i_*exhibited the greatest uncertainty. NEXI showed more bias, systematically underestimating*t_ex_*and*f*, and overestimating*D_i_*.

#### 3.3.2 Reproducibility

**Fig. 5.**
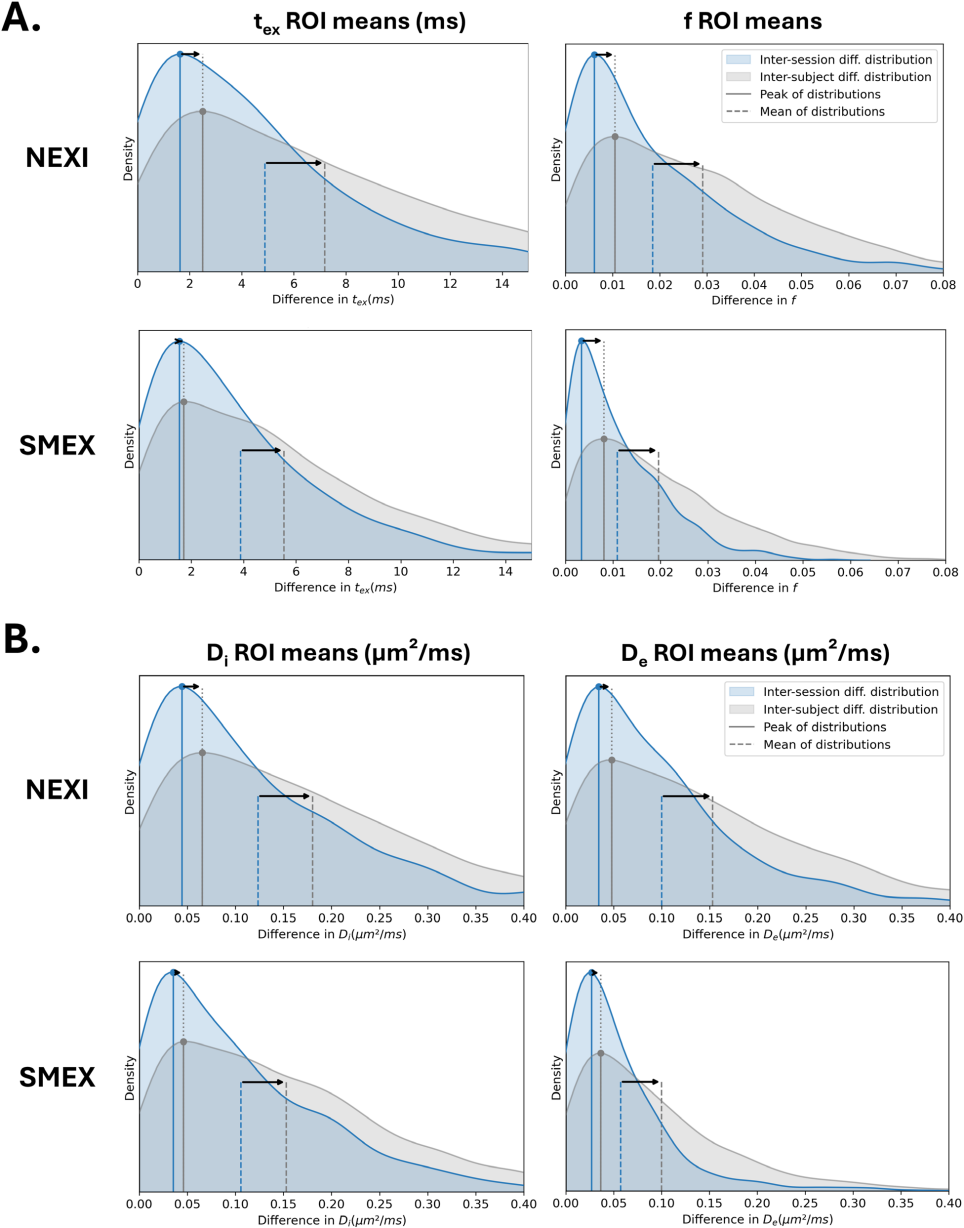
(A) Distributions of the absolute differences of the DKT ROI means of NEXI and SMEX*t_ex_*and*f*estimates between two sessions of the same subjects (blue) and between pairs of subjects (gray). (B) Equivalent distributions for*D_i_*and*D_e_*estimates. The modes/peaks seem to vary less between inter- and intra-subject distributions, the difference being more in the tail of the distribution, which shifts the overall mean of the differences.

To assess intra-subject variability, we compared the first and second sessions of all our subjects for each DKT ROI and plotted the distribution of these differences. To assess inter-subject variability, we compared the first session of each subject with those of the others. We show these distributions for*t_ex_*and*f*(Fig. 5A) and for the diffusivities (Fig. 5B) for NEXI and SMEX. Additionally, to provide information on the sign of the scan-rescan variability, Bland-Altman plots for the inter-session comparisons are provided inSupplementary Figure S8. A Wilcoxon signed-rank test showed that the differences between scans but also between subjects are smaller for SMEX than NEXI (p < 0.0001). SMEX also improves intra-subject correlations compared to NEXI, as shown inTable 2. Both models, however, display modes and means of the estimation difference distributions that are greater for inter-subject differences than intra-subject differences indicating that the models are sensitive to inter-subject variations. The inter-subject correlation is also less pronounced than the inter-session (or intra-subject) correlation (Table 2).

**Table 2.** Correlation coefficients of the DKT ROI means between the scan-rescan sessions of all subjects (denoted intra-subject) and between the different subjects’ first session (denoted inter-subject).

	Correlation type	* t _ex_*	*f*	* D _i_*	* D _e_*
NEXI	intra-subject correlation	0.74	0.78	0.77	0.74
	inter-subject correlation	0.51	0.62	0.63	0.55
SMEX	intra-subject correlation	0.76	0.90	0.81	0.81
	inter-subject correlation	0.49	0.77	0.67	0.61

The inter-subject correlation is less pronounced than the intra-subject one. The SMEX model also shows stronger correlations both intra- and inter-subjects, except for*t_ex_*. All correlations verified p < 0.0001.

### 3.4 Correspondence between NEXI parameters and the MWF

The correlations between each NEXI parameter and the MWF across DKT ROIs are shown inFigure 6. The exchange time shows a significant correlation to MWF (r = 0.74, 0.76, respectively for NEXI and SMEX), followed by the extra-cellular diffusivity*D_e_*(r = 0.41, 0.55), and to a lesser extent*f*(r = -0.37, -0.30) and*D_i_*(r = -0.31, -0.36). The correlation coefficients and p-values are very similar between the two model variants, showing the same trends.

**Fig. 6.**
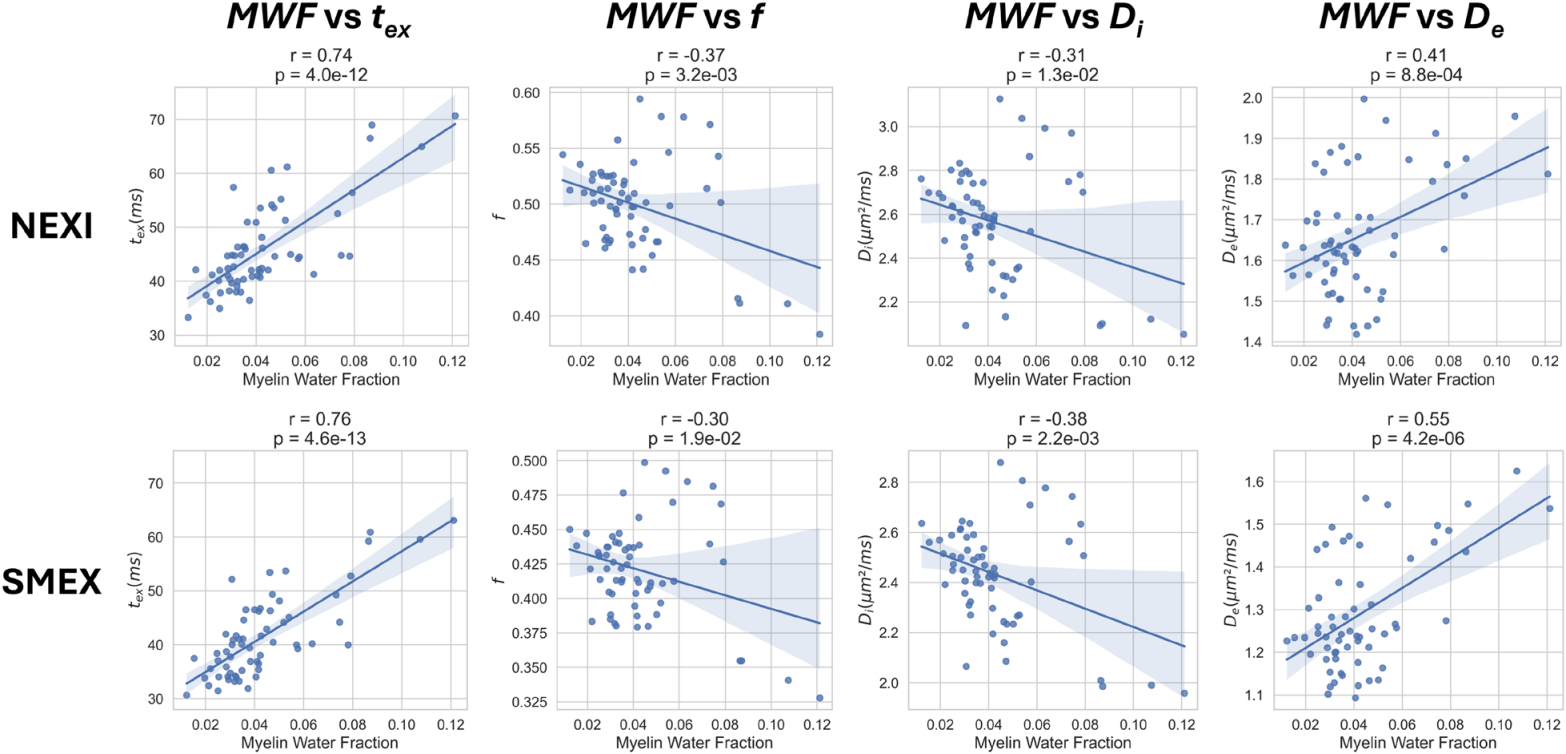
Correlation between average MWF of the DKT ROI and average parameters of NEXI and SMEX. In both NEXI models, there is a very significant strong positive correlation between MWF and*t_ex_*, a moderate positive correlation with*D_e_*, and weak negative correlations with*f*and*D_i_*.

The strong relationship between*t*_ex_and MWF is also reflected in the patterns observed on cortical surfaces projections. MWF and*t*_ex_projections are plotted inFigure 7where we provide the*T_1_w/T_2_w-ratio*Myelin Fraction map from (Glasser et al., 2016) as reference for comparison. Similar patterns of elevated MWF and longer*t*_ex_can be observed in the primary motor cortex and other areas proximal to the central sulcus. However, the higher*t_ex_*patterns in the anterior temporal lobe are not matched in the MWF map, nor in the reference Myelin Map. This indicates that, despite the strong correlation, the MWF is not sufficient to explain the variations in*t_ex_*across the cortex. Overall, the MWF map obtained through T_2_relaxometry and the Myelin Maps show some consistent patterns mostly in the areas round the central sulcus; however, the Myelin map patterns found in the parietal, occipital (V1, MT), and temporal lobe (A1) are not present in the MWF.

**Fig. 7.**
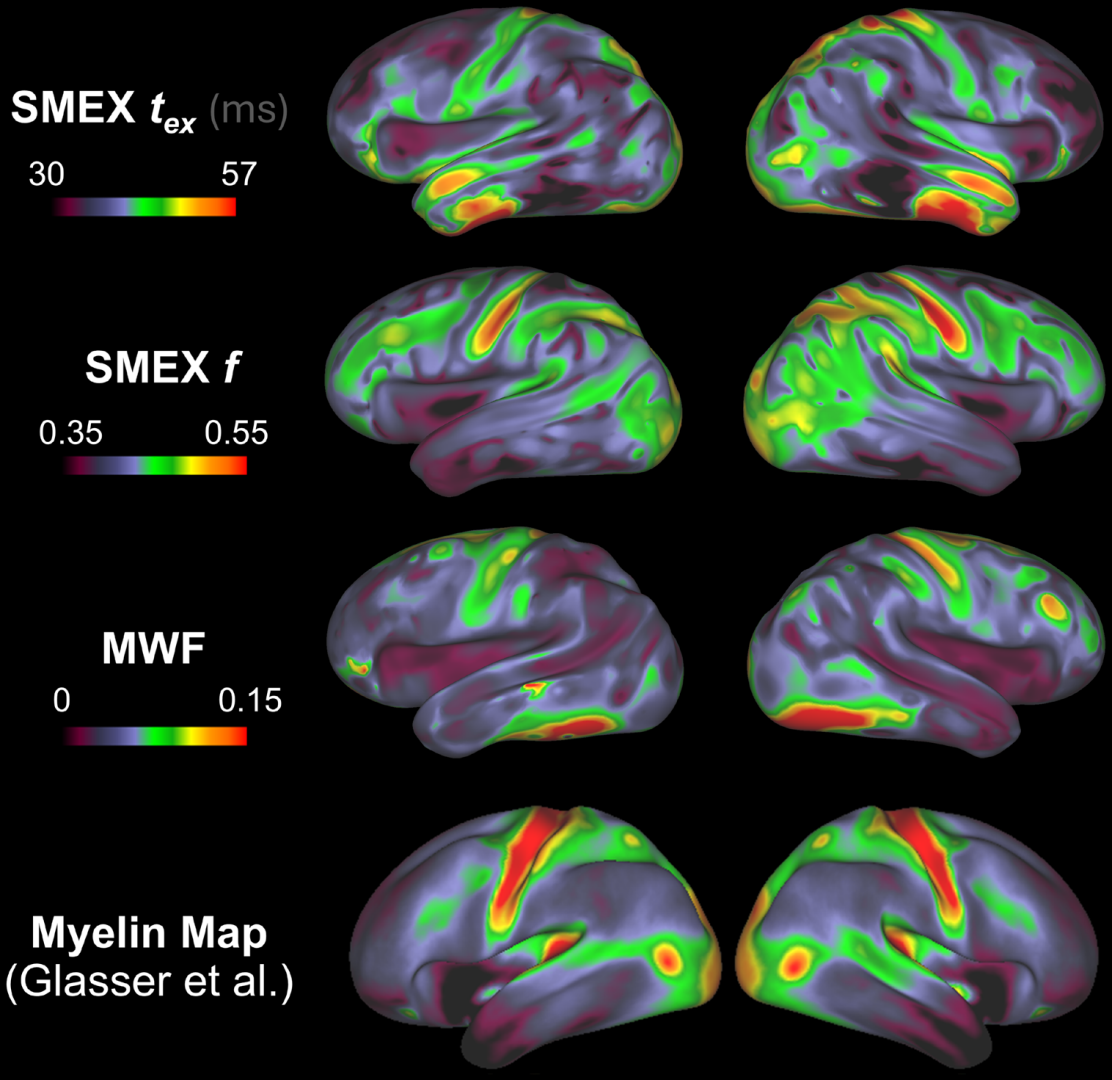
Projection onto cortical surface of SMEX*t_ex_*estimations and the MWF. A reminder of the Cortical Myelin Maps using T_1_w/T_2_w ratio obtained in (Glasser et al., 2016) is shown below for reference. Both the*t_ex_*and MWF maps show higher values in the motor cortex and along the central sulcus, which is also largely present on the Myelin Map. The maps show differences in the temporal lobe.

The MWF shows weaker correlations with the DTI/DKI scalars than with*t_ex_*, with the strongest correlations observed for AK (r = 0.52, p = 2e-05) and AD (r = 0.50, p = 3e-05).

### 3.5 Correspondence with cortical thickness

The correlations between NEXI parameters and cortical thickness across ROIs is shown inSupplementary Figure S9, as well as a comparison of cortical surface maps inSupplementary Figure S10. The intra-neurite fraction shows a significant correlation to cortical thickness (r = -0.74, -0.78, respectively for NEXI and SMEX), closely followed by the intra-cellular diffusivity*D_i_*(r = -0.68, -0.66), and to a lesser extent the extra-cellular diffusivity*D_e_*(r = -0.45, -0.36). Remarkably,*t*_ex_does not correlate with cortical thickness. The patterns of*f*and cortical thickness across the brain surface confirm these two measures to be strongly anti-correlated.

## 4 Discussion

In this study, we present the first in vivo quantification of human cortical microstructure using the NEXI/SMEX models on data acquired with a clinical MRI scanner. In our previous NEXI implementation on Connectom data (Uhl, Pavan, Molendowska, et al., 2024), we established that the model estimates greatly benefitted from accounting for the Rician mean floor in the fitting procedure. One of the open questions of our previous work was how NEXI could translate to clinical systems, given that weaker gradient strength and different stimulation limits needs to be compensated by longer gradient pulse durations to achieve high b-values with short diffusion times. Here we therefore compared two implementations of NEXI: NEXI (Jelescu et al., 2022), which approximates diffusion gradients as instantaneous pulses, and SMEX (Olesen et al., 2022), which integrates the actual gradient waveforms for greater accuracy at the cost of a greater computation time. Both implementations already account for the Rician floor. The Rician noise assumption is supported by the use of the Adaptive Combine algorithm, which better preserves the original noise characteristics of the MRI data of each individual coil channel; in contrast, a conventional sum-of-squares channel combination approach would result in a non-central chi noise distribution (den Dekker & Sijbers, 2014).

While SMEX offers greater accuracy for clinical protocols with long gradient durations, its computational burden remains a limitation. Recent work has shown that this cost can be substantially reduced by using a neural network trained to approximate the SMEX forward model, while still relying on nonlinear least squares for parameter estimation (Olesen et al., 2022). This strategy preserves the interpretability and robustness of the fitting process, as the neural network serves only as a fast signal predictor rather than performing the inversion itself. While our initial results adapting this approach for github.com/Mic-map/graymatter_swissknife appear promising, particularly in terms of speed, further work is needed to systematically evaluate the precision and robustness of this approach under realistic experimental conditions and lower SNR.

The comparison of NEXI and SMEX was based on experimental data in the human cortex, as well as noise propagation in synthetic data. As in (Uhl, Pavan, Molendowska, et al., 2024), we also examined intra- versus inter-subject variability of NEXI estimates. The sensitivity to individual differences is an important feature for clinical translation. Finally, we explored the relationship between NEXI estimates and complementary measures, including correlations with MWF and cortical thickness, to provide additional context for their interpretation.

First, our findings support the suitability of NEXI/SMEX to model diffusion MRI data acquired in human cortical gray matter on a clinical scanner. Indeed, in spite of the more limited diffusion time range that can be probed, and the more modest b-values achievable for each diffusion time, the data in gray matter displayed the expected signature feature of significantly lower signal with longer diffusion times, which was detectable at individual subject and ROI-level. Although the dynamic range of signal change between the extreme diffusion times was modest, it was still more marked in GM than in WM, which further suggests sensitivity to exchange. Increasing the range of diffusion times to increase the dynamic range of signal changes in GM would come at a cost of longer TE (to accommodate longer diffusion times) which may introduce a substantial penalty in SNR.

Consistently, our findings further reveal that both NEXI and SMEX yield parameter estimates in agreement with previous studies on human gray matter using the Connectom scanner (Uhl, Pavan, Molendowska, et al., 2024) and in vivo rat cortex (Jelescu et al., 2022). Cortical features previously quantified on the Connectom scanner were, on average:*t_ex_*= 42 ms,*f*= 0.38,*D_i_*= 3.35 µm²/ms,*D_e_*= 0.92 µm²/ms, which is largely similar to values reported here inTable 1. Notably,*t_ex_*consistently falls within the range of tens of milliseconds.

While the overall spatial patterns of estimated NEXI parameters across the brain remain consistent between the two variants, SMEX, with its more precise gradient integration, tends to produce lower mean parameter values compared to NEXI, and largely resolves the bimodality of solutions across all cortical voxels, particularly for*f*and*D*_e_. Surprisingly, the AICc of NEXI is somewhat lower, that is, the fit of this model described the signal better. However, a better fit does not always guarantee a better model, especially as the AICc confidence intervals of the two variants overlap. Indeed, SMEX results seem more plausible from a physical point of view. For example, the distribution of*D*_i_values across all cortical voxels displays fewer extreme values (higher than the free water diffusivity at 37 °C), resulting in a mean*D*_i_value over DKT ROIs of 2.45 µm^2^/ms, which is similar to axonal diffusivity reported in white matter (Dhital et al., 2019;Howard et al., 2022;Tristão Pereira et al., 2021).

One possible explanation for this discrepancy is technical: the estimation of the Jacobian matrix during SMEX fitting is computationally more demanding, and small inaccuracies in its calculation may prematurely halt convergence or bias the optimization process. Alternatively, the result may reflect limitations in the model itself, such as the assumption of Gaussian diffusion in each compartment or the absence of a soma compartment, which could have a stronger impact on model accuracy in real tissue. Further investigation is needed to disentangle these factors. In either case, both NEXI and SMEX provide a better fit to the data compared to the SANDI model. While SANDI is not designed for data with long*t_d_*, attempts have recently been made to apply it to clinical data (Schiavi et al., 2023). Our results indicate that, in the context of a clinical scanner, NEXI and SMEX better describe the data that can be acquired.

Using synthetic data, we examined how NEXI estimates are impacted by long pulses. For all parameters, NEXI estimates display larger bias and poorer precision vs. the ground truth as compared with SMEX estimates. These results are not observed when δ is reduced to 4 ms. In this scenario, NEXI significantly reduces its bias and uncertainty for all parameter estimates with the exception of*D*_i_, while SMEX maintains biases and variances similar to those seen with longer δ. This suggests that SMEX bias may stem from the estimator itself, whereas NEXI bias likely arises from the violation of the short-pulse assumption.

The discrepancy between NEXI and SMEX estimates, particularly in the exchange time, raises questions about the validity of the narrow pulse approximation in clinical settings with longer gradient durations. While NEXI offers a computationally efficient approach for parameter estimation, its accuracy may be compromised as shown by the simulation. SMEX, on the other hand, provides a more accurate representation of the diffusion process but at the cost of increased computational complexity. Neural networks are a promising approach to reduce the computation time for SMEX. A comparative study evaluating the performance of a well-trained neural network against NLS methods in terms of AICc on independent datasets, both synthetic and experimental, would be highly informative. We look forward to seeing developments in this area that do not sacrifice accuracy for speed.

The differences in inter-subject and intra-subject correlations suggest that both models can be sensitive to inter-subject differences. With SMEX, the distinction between inter- and intra-subject differences is less pronounced. While intra-subject correlations are expected to be as high as possible (and they are higher with SMEX than with NEXI), expected inter-subject correlations are difficult to estimate. It is, therefore, possible that one or other of the inter-subject correlations between NEXI and SMEX is the most accurate.

Our analysis reveals a significant positive correlation between*t_ex_*derived from both NEXI and SMEX and the MWF, characterized by a very high correlation coefficient of 0.7. The exchange time is the NEXI parameter that correlates most strongly with the MWF. This correlation aligns with the expectation that regions with higher myelin content exhibit longer exchange times due to reduced membrane permeability. This correlation is also stronger than that with any DKI-derived parameters, highlighting that potentially higher specificity of the relationship between exchange time and myelin as compared to signal representations. The presence of myelin acts as a barrier to water diffusion, hindering the exchange of water molecules between the intra- and extra-neurite compartments. This is supported by previous studies that have shown a relationship between myelin content and restricted diffusion in white matter (Dhital et al., 2019;Lampinen et al., 2020). Spatially,*t_ex_*maps indeed displayed patterns of slower exchange where the Myelin Map showed increased myelin density (Glasser et al., 2016). These patterns were localized around the central sulcus (primarily motor M1 but also somatosensory S1) and in other primary areas such as visual V1 and auditory A1, known for their higher myelin content due to large sensory projections (Glasser et al., 2014;Nieuwenhuys & Broere, 2017), and high-functioning visual areas like middle temporal visual area MT (Born & Bradley, 2005;Glasser et al., 2014;Sánchez-Panchuelo et al., 2012;Sereno et al., 2013).

On the other hand, the anterior temporal lobe showed slower exchange with SMEX while the MWF map showed lower myelin density, similarly to cytoarchitectonic studies also reporting lower myelin content in the area (Glasser et al., 2014;Shafee et al., 2015). This suggests that factors beyond myelination also contribute to variations in exchange time.

The high*t*_ex_values found in the temporal areas are surprising, especially as they do not seem to be explained by myelin content nor partial volume with subcortical white matter (as the cortex is, in fact, particularly thick in the temporal lobe). The correlation between*t_ex_*and MWF could also be influenced by the density of other cell types in the cortex, such as astrocytes, whose processes likely contribute to the “neurite” compartment. This potential influence of other cell types is supported by previous findings in rat brain, where NEXI maps for*f*showed a strong correlation with neurofilament (NeuF) staining, indicating neurite density, in cortical layers, but surprisingly aligned more closely with astrocyte density GFAP staining in hippocampal sub-fields (Jelescu et al., 2022). The density of dendritic spines may also influence the estimate of exchange time across the cell membrane, as the spines may act as a different environment that is in “exchange” with the core of the dendrite (Chakwizira et al., 2024;Şimşek & Palombo, 2024).

The central sulcus, characterized by both high*f*,*D_i_*and low cortical thickness, exemplifies the potential influence of partial volume effects on NEXI parameter estimation. The thinner cortex in this region may lead to increased partial volume with white matter, which could affect the estimates. Since WM is characterized by long impermeable myelinated fibers, the presence of white matter in the same voxel as gray matter could lead to higher estimates of*t_ex_*,*f*(and possibly*D_i_*), as well as MWF. Remarkably, across the whole cortex, the DKT means of*f*and*D_i_*strongly correlate with the cortical thickness, but not*t*_ex_nor MWF. This indicates once again that PVEs, and hence the contribution of WM and therefore myelin to the voxel, are not sufficient to explain changes in*t_ex_*. The significant correlation between cortical thickness and NEXI parameters could also be investigated using a surface-based analysis, which may be more sensitive to local variations than a region-of-interest-based analysis.

In addition to the factors mentioned above, the estimation of NEXI parameters may also be influenced by other microstructural features not explicitly modeled by NEXI, such as the presence of non-Gaussian diffusion within compartments due to irregularities like dendritic spines and neurite beading (Henriques et al., 2019;Lee et al., 2020). Future studies should investigate the impact of these features on NEXI parameter estimation and explore the potential of incorporating them into the model to improve its accuracy and specificity. Such a model has been developed recently (Novikov et al., 2023) but has not yet been applied to clinical nor preclinical data. The inclusion of a soma compartment, as proposed in the SANDIX model (Olesen et al., 2022) or GEM model (Uhl, Pavan, de Riedmatten, et al., 2024), could also be explored to account for the contribution of cell bodies to the diffusion signal in gray matter. However, the inclusion of additional compartments will increase the complexity of the model and make it more challenging to estimate its parameters with sufficient accuracy and precision. The choice of the appropriate model complexity should be guided by the specific research question and the available data quality.

Beyond these limitations, accurate estimation of the noise standard deviation is crucial for Rician noise correction. MP-PCA, while used for this purpose, can introduce biases due to spatial noise correlations common in clinical MRI (Henriques et al., 2023). While our previous work showed that an error on the estimation of σ did not impact the NEXI estimates too much (Uhl, Pavan, Molendowska, et al., 2024), more recent methods that account for the noise correlation and thus yield a more accurate σ map should be considered in future work (Henriques et al., 2023).

Furthermore, the high values of*D_i_*observed in our study, at times exceeding the water diffusion coefficient at body temperature, suggest that the model may be overestimating this parameter. This finding was already noted in our previous Connectom study, although the SMEX model tends to mitigate it (Uhl, Pavan, Molendowska, et al., 2024). This parameter is notoriously difficult to estimate for all models (Howard et al., 2022;Jelescu, Veraart, et al., 2016;Palombo et al., 2020). This could be due to several factors, including the presence of noise, partial volume effects, and the limitations of the model in capturing the complex diffusion environment within neurites. Future studies should investigate these factors in more detail and explore methods to improve the accuracy of*D_i_*estimation, such as trying more realistic models of neurite geometry and diffusion. The assumption of uniaxial diffusion within neurites may not hold in regions with complex neurite architectures, due to the presence of dendritic spines and other neurite irregularities that are not captured by the current NEXI model. As for the*D_i_>D_e_*inequality displayed in our results, we note that it is only a soft constraint in our fitting implementation. It does guide the initial parameter search process, ensuring*D_i_>D_e_*during the grid search phase. However, it is important to note that, after the grid-search, the parameters are allowed to adjust more freely, which can occasionally result in*D_i_<D_e_*. While the*D_i_>D_e_*estimate holds for most voxel estimates here, it is noteworthy that on postmortem human data, this was not typically the case (in spite of similar fitting procedure) (Hertanu et al., 2023).

The comparison of NEXI parameter estimates with histological data is crucial for validating the model and interpreting its parameters in terms of underlying tissue microstructure. However, such comparisons are challenging due to the limited availability of high-resolution histological data in the human brain. Future studies will aim to acquire high-quality histological data in conjunction with NEXI measurements on postmortem human brains to establish a more direct link between model parameters and tissue microstructure (Hertanu et al., 2023).

For the NEXI model to be successfully implemented in clinical practice, reducing the acquisition time is crucial. The present 27-min protocol is too lengthy for routine scans. A streamlined, shorter protocol would allow for the efficient collection of data from patients with neurological conditions, potentially enhancing diagnostic capabilities. Consequently, a key objective of our ongoing research is to develop a faster protocol without compromising the quality of the NEXI parameter estimation (Uhl et al., 2023,^2025^).

## 5 Conclusion

This study is the first to demonstrate the feasibility of estimating NEXI parameters in the human cortex in vivo using a clinical MRI scanner. Our findings reveal that NEXI and SMEX provide similar gray matter microstructure parametric maps, with SMEX offering potentially more accurate and biologically plausible results due to its consideration of wide diffusion pulses. The strong correlation between the exchange time and MWF further supports the biological relevance of NEXI/SMEX parameters and the potential of t_ex_as biomarker for cell membrane permeability – whether related to myelination or cell integrity – in the brain.

Future work will focus on shortening acquisition protocols for clinical scanners and exploring the potential of NEXI/SMEX as a biomarker for various neurological conditions. The development of stronger gradients on clinical scanners and advancements in denoising techniques are expected to further improve the accuracy and precision of NEXI estimates, paving the way for its wider clinical application.

## Supplementary Material

Supplementary Material

## Data Availability

The code used in this study is available onhttps://github.com/Mic-map/graymatter_swissknife. The code for the Myelin Water Fraction estimation (Canales-Rodríguez et al., 2021) used in this study is available athttps://github.com/ejcanalesr/multicomponent-T2-toolbox. The data used in this study are available upon request after signing a formal data sharing agreement and providing approval from the requesting researcher’s local ethics committee.
