# Supplementary material for: Human gray matter microstructure mapped using neurite exchange imaging (NEXI) on a clinical scanner
Source: Imaging Neurosci (Camb). 2025 Jun 12;3:IMAG.a.32. doi: 10.1162/IMAG.a.32 (PMC12319888; doi:10.1162/IMAG.a.32)
Supplement: Supplementary Material [file imag.a.32_supp.pdf]

Human gray matter microstructure mapped using Neurite Exchange Imaging (NEXI) on a clinical scanner

Quentin Uhl[1], Tommaso Pavan[1], Thorsten Feiweier[2], Gian Franco Piredda[3,4] and Ileana Jelescu[1]

[1]Department of Radiology, Lausanne University Hospital (CHUV) and University of Lausanne, Lausanne, Switzerland; [2]Siemens Healthineers AG, Erlangen, Germany, [3]Advanced Clinical Imaging Technology, Siemens Healthineers International AG, Lausanne, Switzerland, [4]CIBM Center for Biomedical Imaging, Geneva, Switzerland.

Supplementary Material

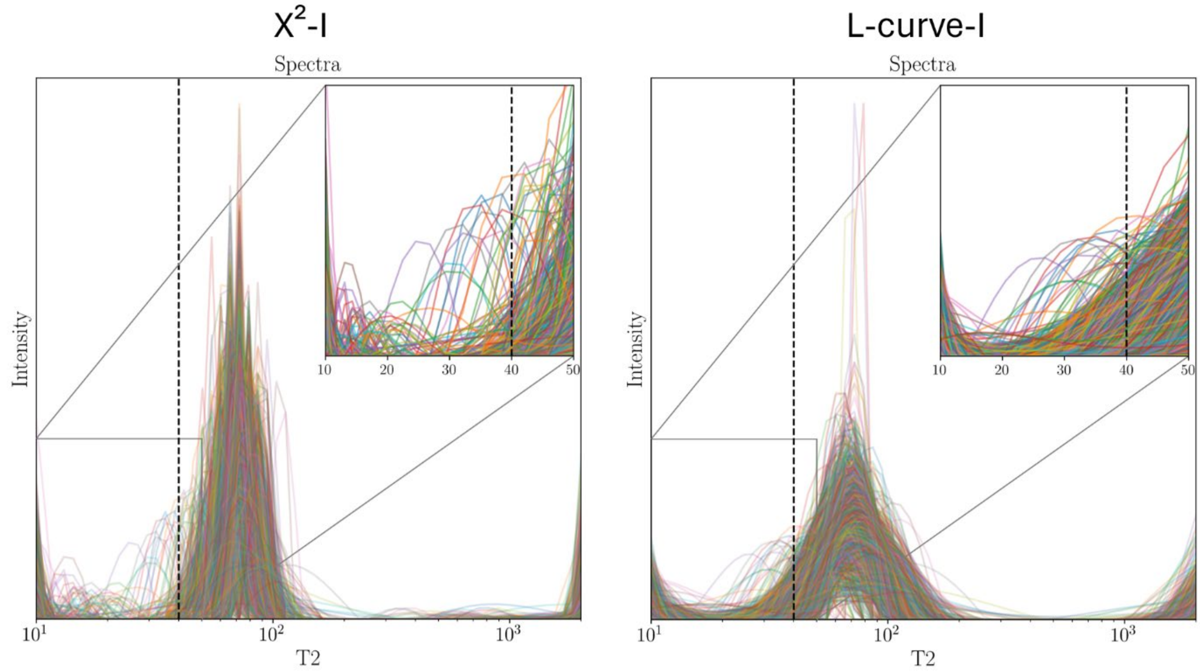

Fig. S1 Comparison of the MWF cutoff of the intensity T_2 spectrum lobes from one subject from χ^2-I and L-curve-I methods. These methods are recommended for MWF extraction respectively for moderate and high level of noise. The lobe separation of the T_2 spectrum reveals that the χ^2-I method is better suited to our data.

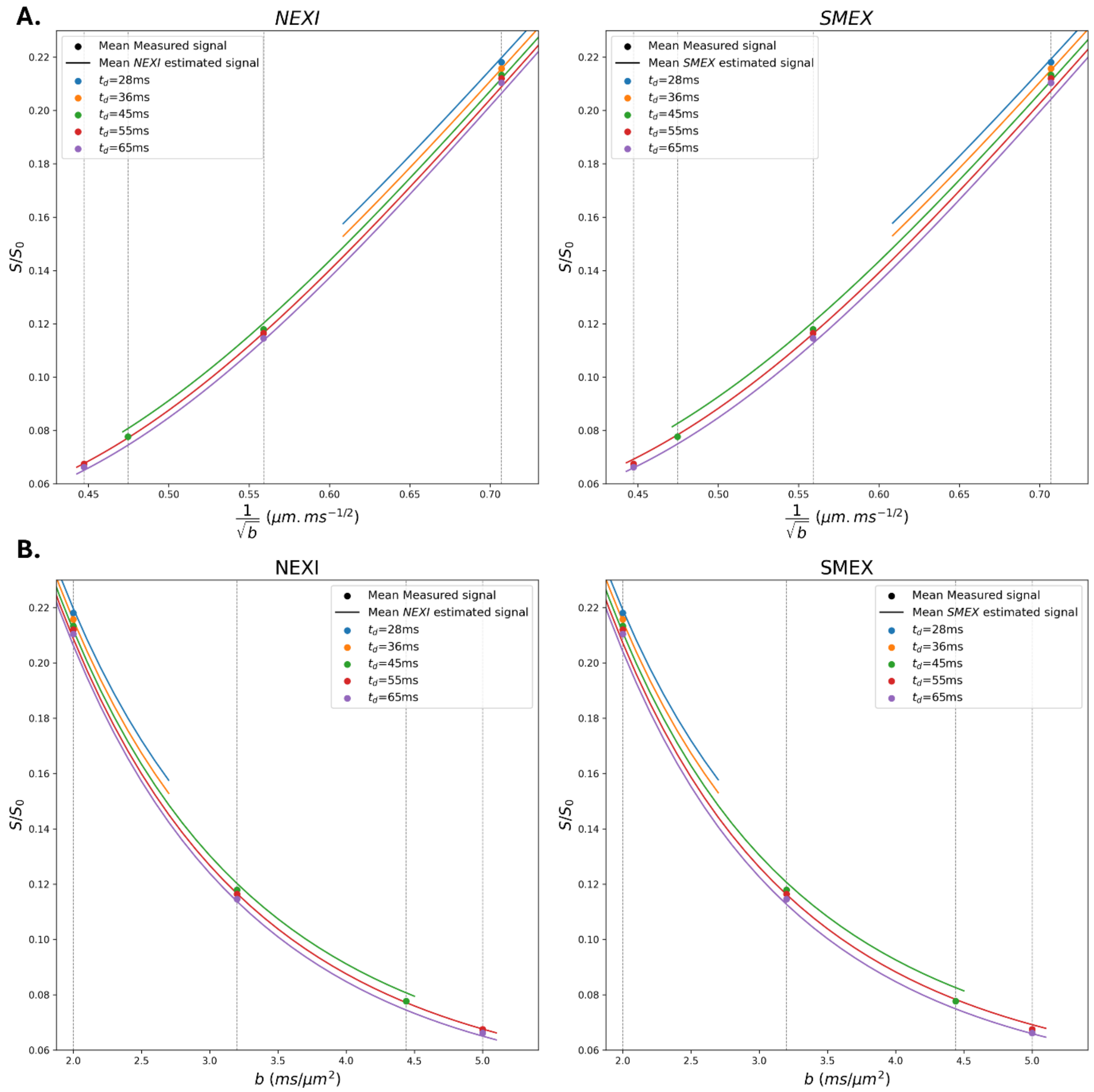

Fig. S2 Mean measured signal, represented by dots, and mean estimated signal curves of the whole cortical ribbon from NEXI and SMEX implementations with respect to $1/\sqrt{b}$ (**A.**) and b (**B.**). At short t_d, the measured signals have lower maximum b-value. Thus, at b=5 ms/µm², on the left on the x-axis, we only have signals from t_d at 55 and 65 ms, at b=4.44 ms/µm², only 45 ms and at b=3.2 ms/µm² we have t_d at 45, 55 and 65 ms. The short t_d curves are therefore only partially shown.

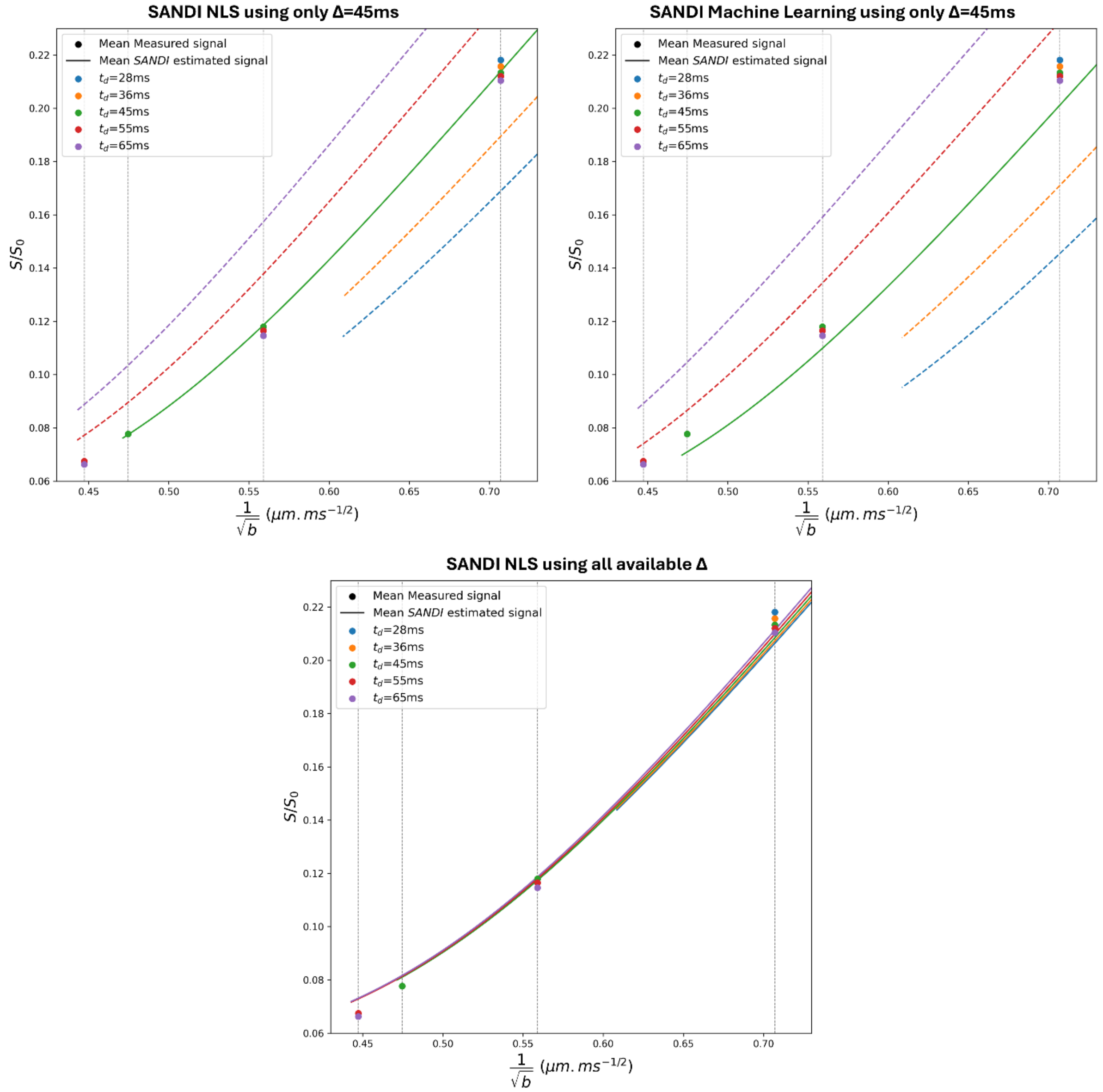

SANDI estimation using:	D_i (μm²/ms)	D_e (μm²/ms)	$f_{neurite}$	f_{soma}	r_s (μm)	AICc
Our python package (NLS) using **Δ=45ms**	**1.85** [1.58 - 2.11]	**2.02** [1.64 - 2.40]	**0.20** [0.14 - 0.26]	**0.10** [0.07 - 0.13]	**14.8** [14.0 - 15.6]	**Δ=45ms:** -89.7 **All Δ:** -47.7
MATLAB Toolbox (ML) using **Δ=45ms**	**1.85** [1.82 - 1.88]	**1.77** [1.49 - 2.05]	**0.09** [0.05 - 0.15]	**0.49** [0.38 - 0.58]	**13.9** [13.2 - 14.6]	**Δ=45ms:** -45.0 **All Δ:** -40.7
Our python package (NLS) using **all Δ**	**2.90** [2.52 - 3.27]	**0.94** [0.71 - 1.29]	**0.20** [0.14 - 0.26]	**0.10** [0.07 - 0.13]	**23.6** [20.6 - 26.6]	**All Δ:** -81.5

Fig. S3 Mean SANDI model fitting across all subjects, along with the modes and confidence intervals of SANDI parameter estimates within DKT ROIs. Results are shown for two data configurations: using all available diffusion weightings and using only the 5 b-values at Δ = 45 ms. The mode and confidence intervals of the corrected Akaike Information Criterion (AICc) are also provided for each fit. For models utilizing only the Δ = 45 ms data, predicted curves and AICc values for the other Δ values are included for illustrative purposes, highlighting the importance of varying diffusion time. In all cases, the SANDI model fit does not fully capture the signal decay trend with increasing diffusion times. While the Machine Learning (ML) model from the Matlab toolbox yields similar diffusivities and radii compared to the Non-Linear Least Squares (NLS) approach, it produces substantially different volume fractions. Notably, in all three cases, the neurite fraction estimate is unrealistically low and the soma radius too large.

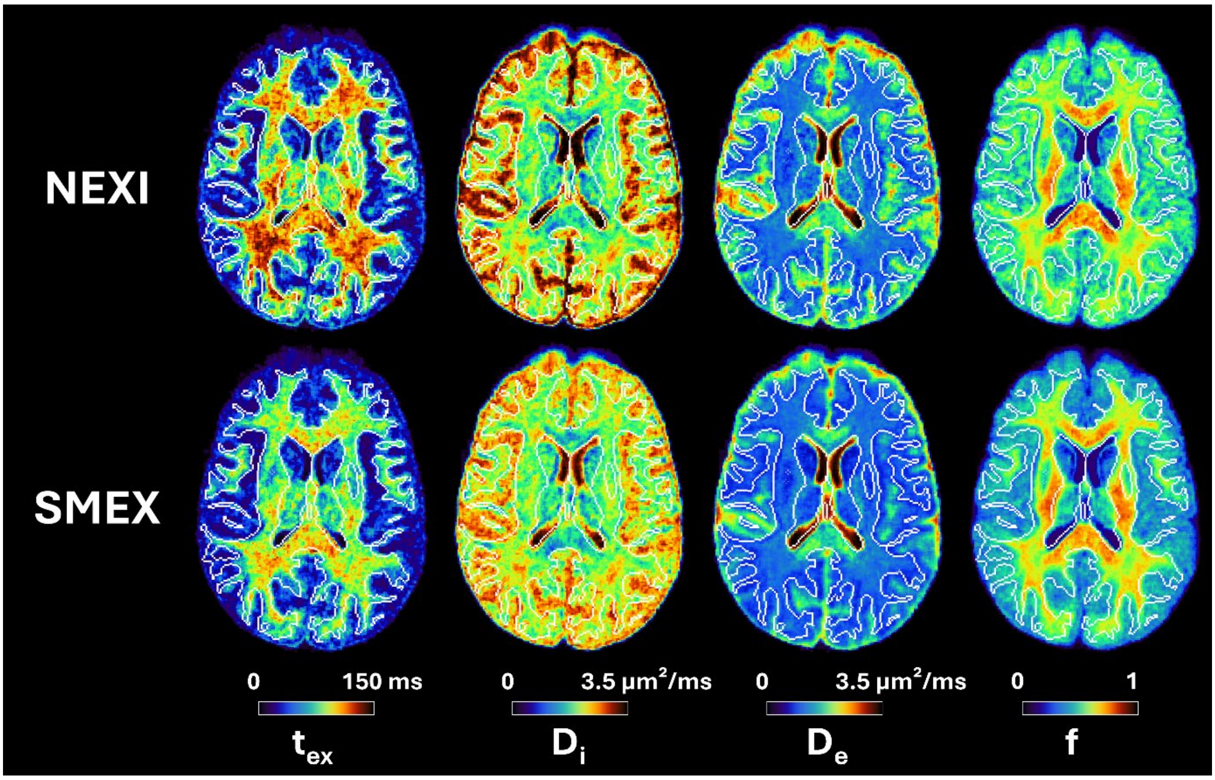

Fig. S4 Axial slice of NEXI and SMEX parametric maps, averaged across sessions and subjects (N = 22). The white matter is indicated by the white outlines. t_{ex} and D_e are consistent throughout the cortex. f and t_{ex} display the expected anatomical pattern in white versus gray matter. However, the white matter estimates are unreliable as they deviate from the NEXI model assumptions and are also largely influenced by the fit boundaries; they are shown for visualization purposes only. D_i shows large variability across voxels, while hitting its upper bound frequently.

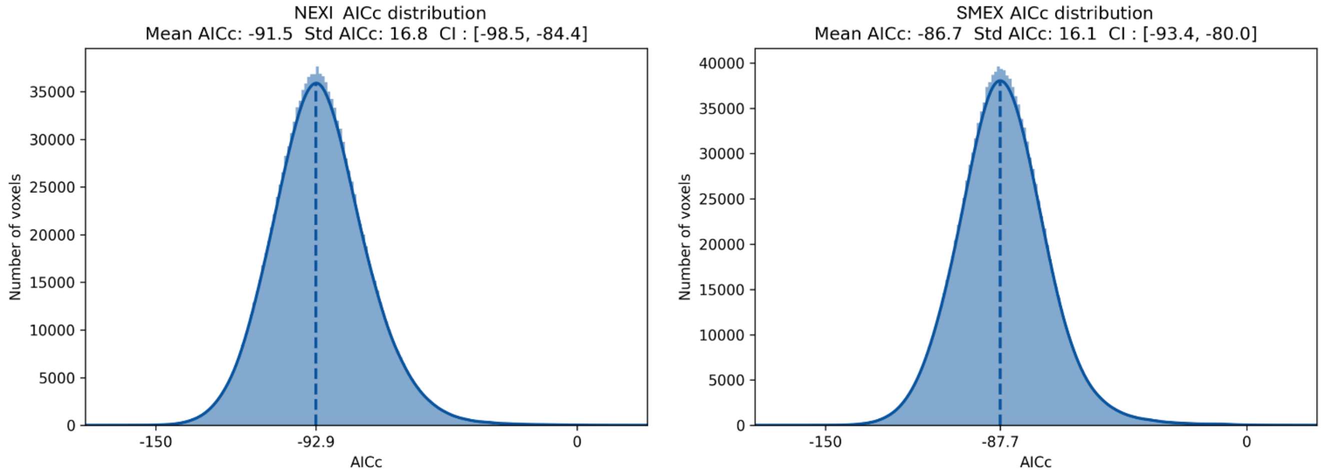

Fig. S5 Corrected Akaike Information Criterion (AICc) distribution of NEXI and SMEX in the whole cortical ribbon. The performance of NEXI and NEXI is comparable, and their confidence intervals overlap.

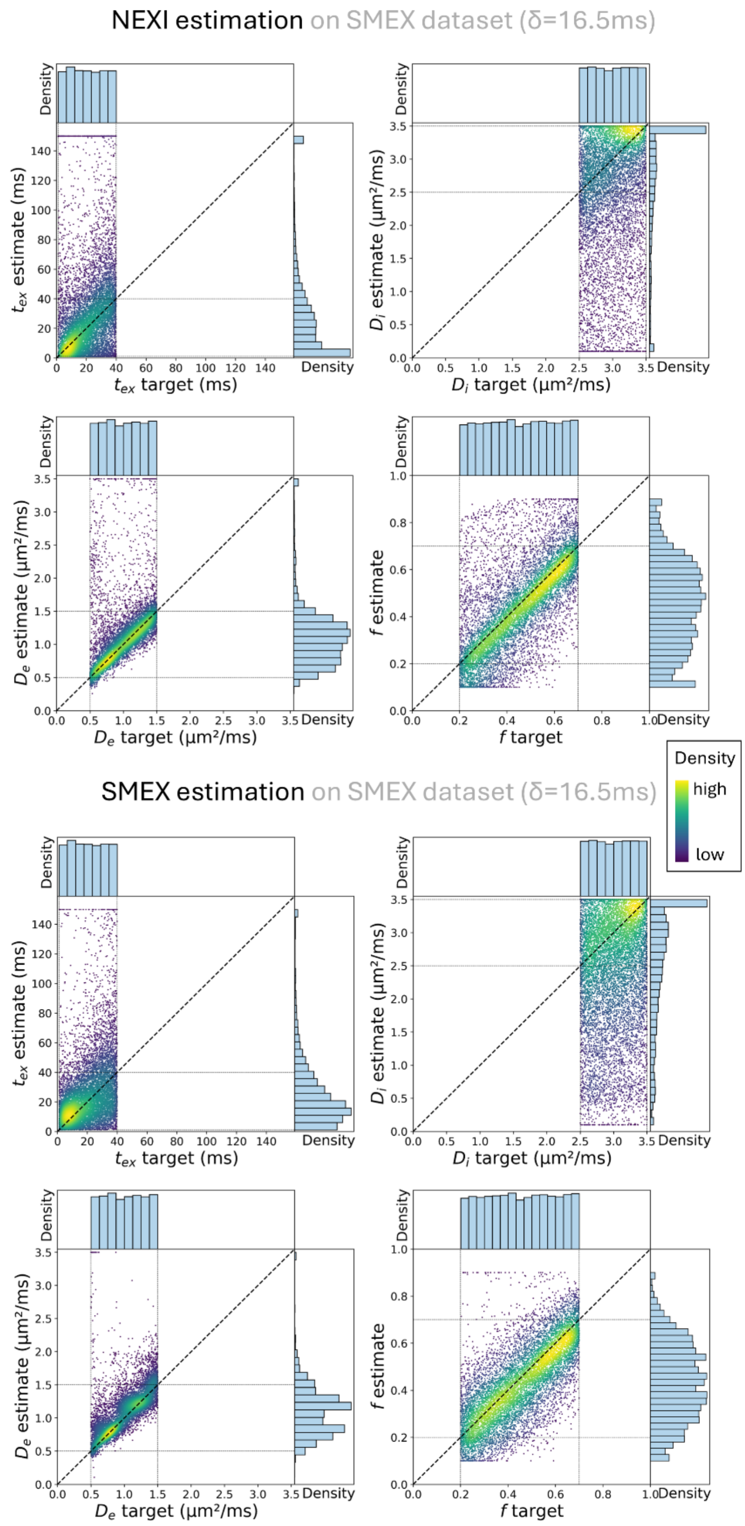

Fig. S6 Scatter plot of NEXI and SMEX parameter estimates from synthetic signals generated using uniformly sampled ground truth parameter values within plausible ranges (t_{ex}: 1–40 ms, D_i: 2.5–3.5 µm²/ms, D_e: 0.5–1.5 µm²/ms, f: 0.2–0.7), and experimental Rician noise levels. Root Mean Square Errors (RMSE) confirm that SMEX yields more accurate estimates for all parameters except Di, for which both models perform similarly. Compared to simulations in Fig. 4 (based on SMEX-derived ground truth), this configuration reduces bias in NEXI estimates, particularly for f and De.

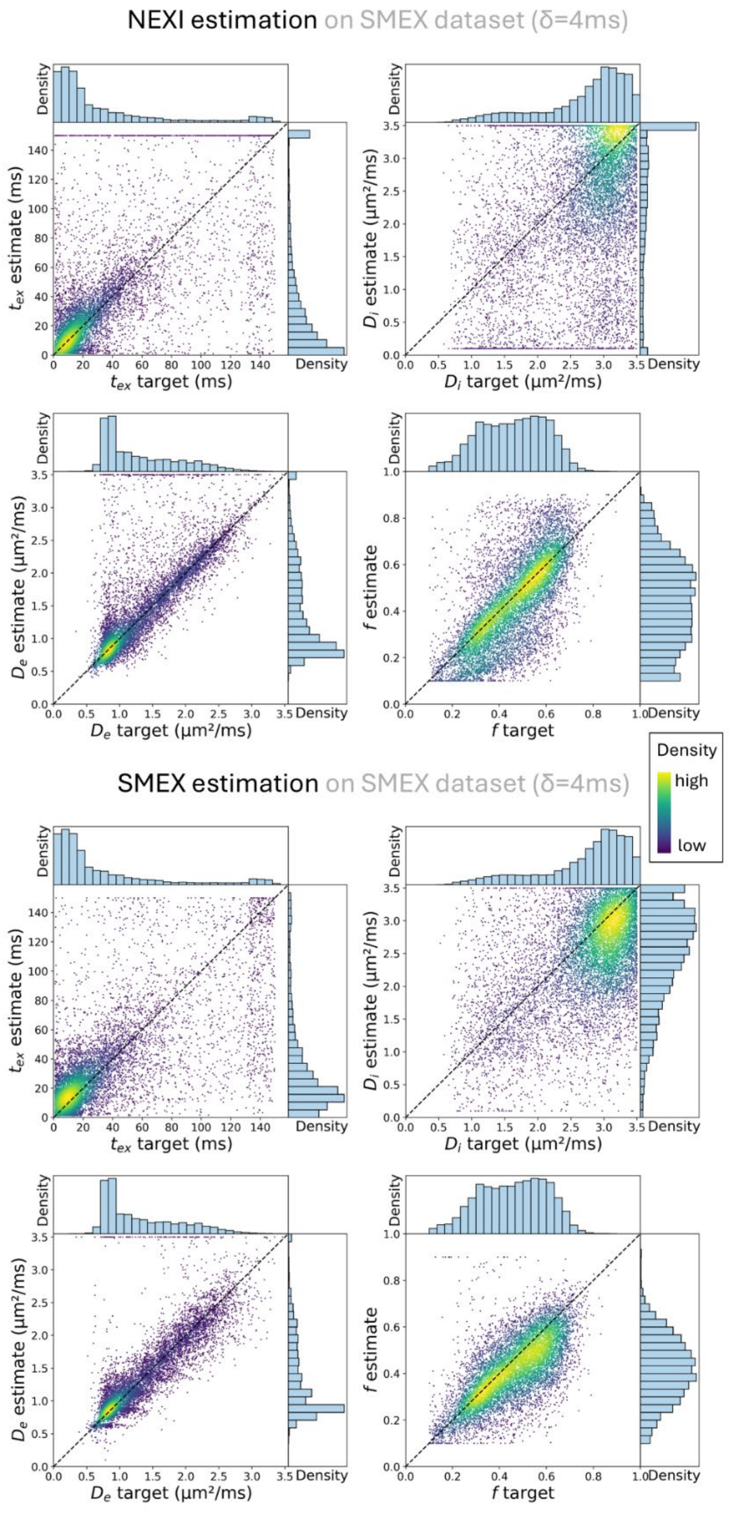

Fig. S7 Scatter plot of NEXI and SMEX parameter estimates from synthetic SMEX signals generated using the experimental estimates from SMEX as ground truth, and experimental Rician noise levels, as in Fig. 3, but setting δ to 4 ms. This time, SMEX showed more bias than NEXI in the estimation of f, somewhat poorer precision in the estimation of t_{ex}, but retains better performance in estimating D_i.

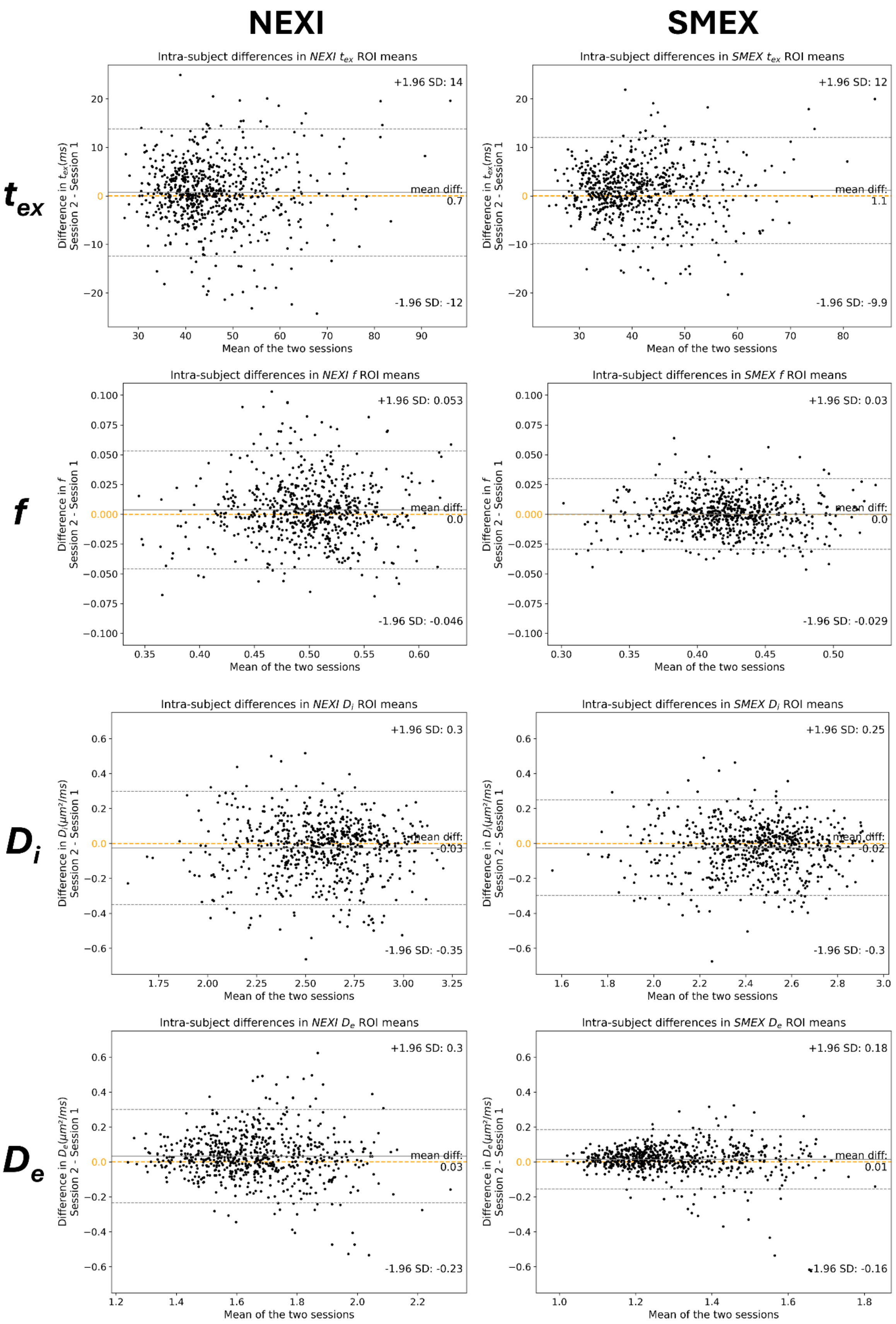

Fig. S8 Bland-Altman plots of the DKT ROI means of NEXI and SMEX estimates showing scan-rescan repeatability across subjects. The mean difference is close to zero for all the parameters of each model. The confidence interval is reduced with SMEX vs NEXI.

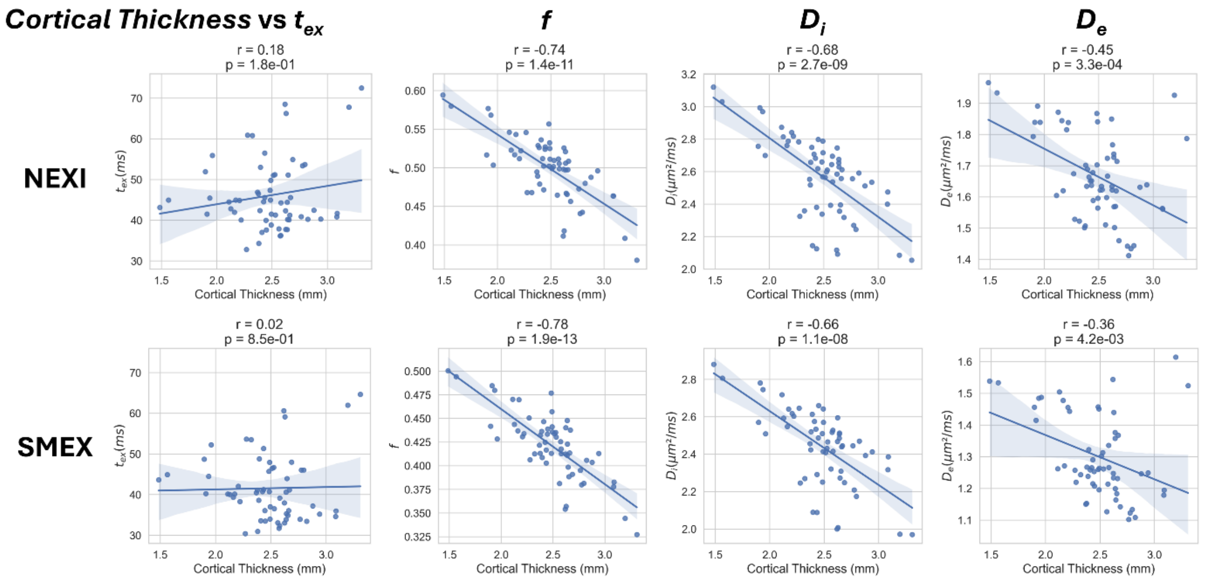

Fig. S9 Correlation between DKT ROI means of Cortical Thickness and NEXI parameters in both NEXI and SMEX implementations. There is a very significant and strong negative correlation between cortical thickness and f as well as with D_i.

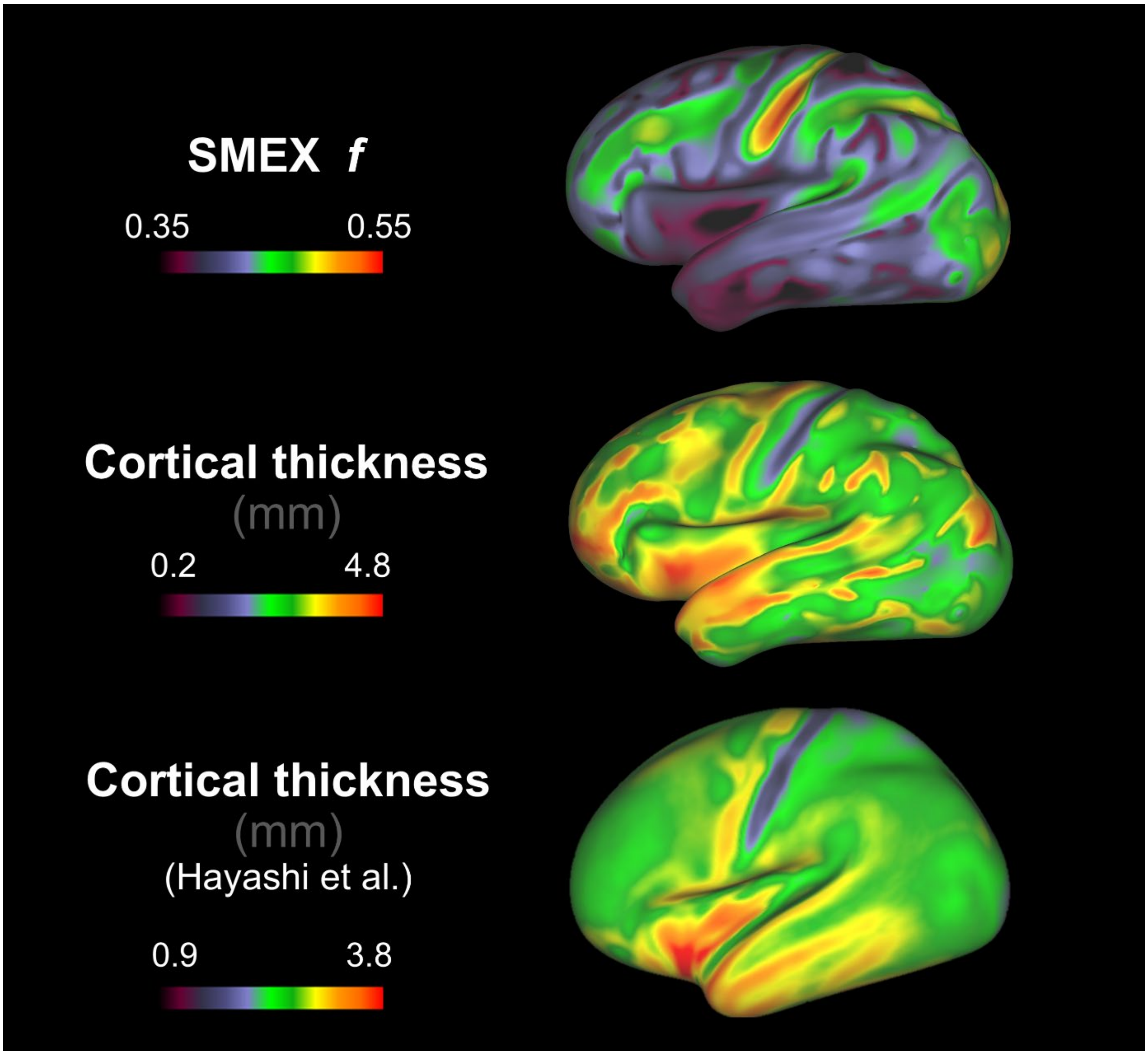

Fig. S10 Projection onto cortical surface of SMEX *f* estimations and the cortical thickness. A reminder of the cortical thickness maps obtained in (Hayashi et al., 2021) is shown below for reference. The maps show similar trends. Especially, the main decrease in cortical thickness around the central sulcus is matching with the main increase in *f* in this region.

References

Hayashi, T., Hou, Y., Glasser, M.F., Autio, J.A., Knoblauch, K., Inoue-Murayama, M., Coalson, T., Yacoub, E., Smith, S., Kennedy, H., Van Essen, D.C., 2021. The nonhuman primate neuroimaging and neuroanatomy project. NeuroImage 229, 117726. https://doi.org/10.1016/j.neuroimage.2021.117726
